# Regnase-2 inhibits glioblastoma cell proliferation

**DOI:** 10.1038/s41598-024-51809-x

**Published:** 2024-01-18

**Authors:** Weronika Sowinska, Mateusz Wawro, Jakub Kochan, Aleksandra Solecka, Jarosław Polak, Borys Kwinta, Aneta Kasza

**Affiliations:** 1 Department of Cell Biochemistry, Faculty of Biotechnology, Biochemistry and Biophysics, Jagiellonian University, Krakow, Poland; 2 Department of Neurosurgery and Neurotraumatology, Jagiellonian University Medical College, Kraków, Poland

**Keywords:** Biochemistry, Cancer, Cell biology, Molecular biology, Neuroscience

## Abstract

Regnase-2 (Reg-2/MCPIP2/ZC3H12B) is uniquely expressed at a high level in the healthy brain and down-regulated in samples from patients with glioma, reaching the lowest level in high-grade glioblastoma multiforme (GBM). This RNase is involved in the regulation of neuroinflammation through the degradation of *IL-6* and *IL-1* mRNAs, key pro-inflammatory cytokines for GBM pathology. Reg-2 is a strong inhibitor of the proliferation of human glioblastoma cell lines and blocks their potential to form colonies. Here, we describe that overexpression of Reg-2 stalls glioblastoma cells in the G1 phase of the cell cycle and reduces the level of transcripts implicated in cell cycle progression. These newly identified targets include *CCND1, CCNE1, CCNE2, CCNA2, CCNB1*, and *CCNB2*, encoding the cyclins as well as *AURKA* and *PLK1*, encoding two important mitosis regulators*.* By RNA immunoprecipitation we confirmed the direct interaction of Reg-2 with the investigated transcripts. We also tested mRNA regions involved in their interaction with Reg-2 on the example of *CCNE2*. Reg-2 interacts with the 3’UTR of *CCNE2* in a dose-dependent manner. In conclusion, our results indicate that Reg-2 controls key elements in GBM biology by restricting neuroinflammation and inhibiting cancer cell proliferation.

## Introduction

Glioblastoma multiforme (GBM) is one of the most aggressive malignancies, accounting for 14.5% of all tumors of the central nervous system and 48.6% of malignant tumors of the central nervous system^1^. It is characterized by its highly proliferative index, aggressive invasiveness, and short patient survival (median survival of 15 months)^2^. One of the hallmarks of GBM is neuroinflammation. The inflammatory microenvironment promotes tumor development, migration, invasion, proliferation, resistance to apoptosis, and the maintenance of stem cell-like properties^3,4^. Inflammatory signaling drives the progression of GBM through the continuous secretion of pro-inflammatory cytokines and factors that attract and activate glioma-associated microglia/macrophages. In GBM this feedforward loop relies on the activation of RelB/p50 complexes by the canonical NF-κB pathway resulting in activation of proinflammatory genes. The same pathway inhibits the expression of proinflammatory genes in non-transformed astrocytes^5^.

Regnase-2 (Reg-2/MCPIP2/ZC3H12B) is a recently described RNase involved in the regulation of neuroinflammation^6,7^. It belongs to the Regnase/MCPIP/ZC3H12 family of proteins that includes three other members: Reg-1/MCPIP/ZC3H12A, which is the founder and the best described member of the family, Reg-3/MCPIP/ZC3H12C, and Reg-4/MCPIP4/ZC3H12D. They all possess the conservative nucleolytic NYN/PIN domain and CCCH-zinc finger, both involved in their RNase activity. Reg-2 is present almost uniquely in the brain. It controls brain homeostasis through active regulation of inflammation in primary human and mouse astrocytes and primary mouse microglia^7^. Using the mouse model of sterile neuroinflammation induced by peripheral administration of LPS and a mouse model of multiple sclerosis (experimental autoimmune encephalomyelitis (EAE)) we have shown that Reg-2 level is down-regulated during neuroinflammation. An inflammatory environment reduces the amount of Reg-2 both at mRNA and protein levels. Following IL-1 stimulation, Reg-2 is phosphorylated, ubiquitinated, and degraded by the proteasome^7^. What is more, according to the *Oncopression* database, the Reg-2 level is significantly down-regulated in brain cancer tissue compared to normal brain tissue^8^. We have found that the level of Reg-2 is reduced in human glioblastoma samples, reaching the lowest amount in patients with high-grade glioblastoma multiforme (GBM). A low level of Reg-2 correlates with poor survival of GBM patients^7^. By screening The Cancer Genome Atlas (TCGA) and the Chinese Glioma Genome Atlas (CGGA) databases and their validation using Cox regression analysis, Reg-2 was identified as one of the predictor factors of the prognosis of glioma^9^.

Reg-2 is detected in the cytoplasm where it forms characteristic small, granule-like structures which are typical for the proteins involved in the mRNA turnover^6,10^. Through the direct interaction with the stem-loop structure present in the 3’UTR of interleukin-6 (IL-6) mRNA Reg-2 restricts its level^6,7^. So far, only a few other targets for Reg-2 have been described. They include mRNAs coding another pro-inflammatory cytokine interleukin-1 (IL-1), apoptotic factor IER-3, and a member of the Regnase/MCPIP/ZC3H12 family RNase Reg-1^6,7^. Importantly, both interleukins identified so far as Reg-2 targets are well-described crucial players in GBM biology. Using the TCGA database and patient-derived primary glioma cells, it was established that the expression of IL-6 or IL-1 (and their receptors) is a predictor of an unfavorable prognosis^5,11^. Therefore Reg-2 controls the turnover of transcripts of the cytokines which are significantly correlated with poor glioma patient survival. Recently we have discovered that Reg-2 inhibits the proliferation of human glioblastoma cell line U251-MG and mouse glioblastoma cell line KMWT1 established from the spontaneous glioma tumor^7^. Despite the significant effects of Reg-2 on glioma cells the proliferation mechanisms by which this phenotype is reached remain elusive. Here we reveal the details of Reg-2-dependent inhibition of cell proliferation.

## Results

### Reg-2 inhibits the proliferation of human glioblastoma cell line U87-MG

**Figure 1:**
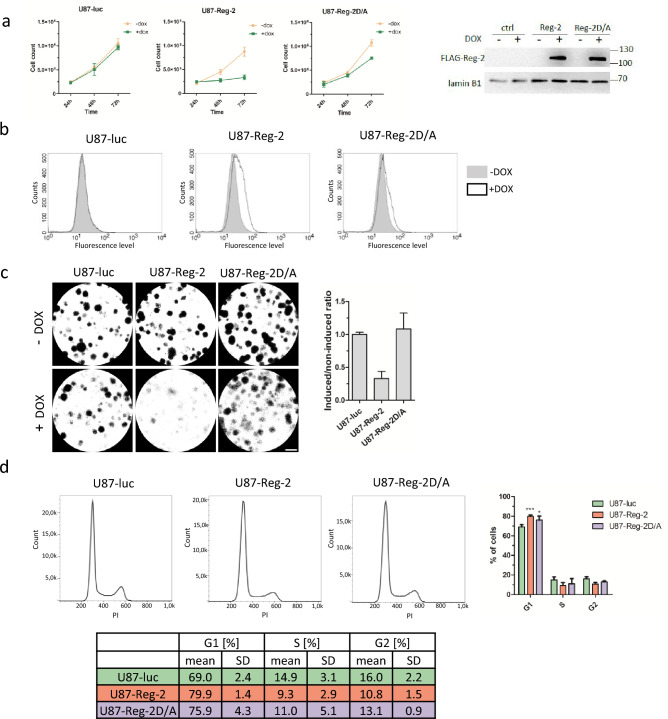
Regnase-2 inhibits proliferation, and stalls the cell cycle at the G1 phase. (**a**) U87-MG cells were treated with dox (1 μg/mL) or left untreated and counted 24, 48, and 72 h after the addition of **d**ox. The levels of Reg-2 and Reg-2 D/A were examined using western blotting. The graph shows results from three independent experiments + / − SD. (**b**) U87-luc, Reg-2, or Reg-2D/A cells were labeled using CellTrace Far Red reagent and divided into two dishes. One dish was induced with doxycycline (1 μg/ml), the other was left untreated. After 48 h of induction, the fluorescence was measured by flow cytometry. The graphs show histogram overlays of noninduced (gray) and induced (white) cells. Representative results of three independent experiments are shown. (**c**) U87-MG cells were plated on 6-well plates (100 cells/well), and treated with dox (1 μg/ml) daily until visible colonies were formed (10 days). Then the colonies were fixed and stained. Graph presents the ratio of the surface covered by colonies formed by dox-induced to non-induced cells normalized to control cells (luc) (mean value of three independent experiments ± SD) (**d**) U87-luc/Reg-2/Reg-2D/A cells were treated with dox (1 µg/mL) for 48 h, and cell cycle analysis was performed. The graphs show a histogram of propidium iodide fluorescence in particular cell lines. Representative results of three independent experiments are shown. The graph and table show the percentages of cells in each cell cycle phase. The data were analyzed using two-way ANOVA and Bonferroni's post-hoc test (***p < 0.001; *p < 0.05).

We have already shown that Reg-2 restricts the proliferation of the U251-MG human glioblastoma cell line and KMWT1 mouse spontaneous glioblastoma cells^7^. To confirm that this phenomenon is universal we analyzed the influence of Reg-2 on another human glioblastoma cell line derived from a malignant glioblastoma tumor, U87-MG. Using the Sleeping Beauty (SB) transposon system we generated U87-MG cell lines with introduced sequences encoding WT Reg-2 (U87-Reg-2) or its mutein Reg-2-D/A (U87-Reg-2D/A) or luciferase (U87-luc) under the control of doxycycline (dox)-inducible promoter. Reg-2-D/A is a nucleolytically inactive protein due to the replacement of one of the crucial aspartic acids in the NYN/PIN domain, required for the binding of magnesium ions, by alanine (D196A)^6,7^. Without dox stimulation, all modified cells divide at the same rate. However, as shown in Fig. 1a the time-dependent increase in the cell number is blocked when WT Reg-2 is expressed. The number of U87-Reg-2 cells remains almost constant within 72 h of their culture in a medium containing dox. Simultaneously, the proliferation of control U87-luc cells under the same conditions remains exponential within the examined period with a doubling time of approx. 24 h. The observed effect of Reg-2 on cell proliferation mainly relies on its nucleolytic activity; however, the number of U87-Reg-2D/A cells after 72 h of dox stimulation is slightly reduced in comparison to untreated and control cells.

Since the detected effect of Reg-2 on cell number could be due to cell death and/or inhibition of their proliferation we employed the CellTrace Far Red assay to monitor cell proliferation. Cells were stained with CellTrace Far Red reagent (Thermo Fisher Scientific) and cultured for 48 h with/without dox. Cell division was tracked by measuring the fluorescent signal generated after the conversion of cell permeable fluorescent molecules to their derivatives by intracellular esterases. As a result of cell division and the spread of fluorescent dye, the signal decreases approximately two-fold per division. Figure 1b shows the shift toward higher intensity of the peak in U87-Reg-2 cells treated with dox. Such an outcome suggests that induction of Reg-2 overexpression results in slower proliferation of U87-Reg-2 cells. The effect of ribonucleolytic inactive mutein (Reg-2-D/A) on U87 cell proliferation is much weaker (Fig. 1b).

To further determine the influence of Reg-2 on cell division we performed a clonogenic assay. This tool allows monitoring in vitro the ability of single cells to grow into a colony. Reg-2 overexpression strongly inhibits glioma cell division and its potential to form colonies (Fig. 1c). This regulation requires an intact NYN/PIN domain as the effect of nucleolytic-inactive Reg-2-D/A on colony formation was similar as in the control cells (Fig. 1c).

### Impact of Reg-2 on the glioblastoma cell cycle

To reveal the mechanisms responsible for Reg-2-dependent inhibition of proliferation, we analyzed its influence on the cell cycle. U87-Reg-2, U87-Reg-2D/A, and control cells were stained with propidium iodide dye, and the number of cells in the individual phases of the cell cycle was estimated by flow cytometry. After dox induction, the population in phase G1 increases from ~ 70% in control to ~ 80% in U87-Reg-2 cells. A similar but much weaker trend is observed in U87-Reg-2D/A cells. The S phase is not significantly affected by Reg-2 overexpression in investigated cells. Cells in the G2 phase accounted for ~ 11% in U87-Reg-2,  ~ 13% in U87-Reg-2D/A, and ~ 16% in control cells (Fig. 1d). We concluded that Reg-2 stalls U87-MG glioblastoma cells in the G1 phase. This process relies mainly on its nucleolytic activity; however, inactive ribonucleolytic Reg-2 also exerts a weak effect.

### Reg-2 targets are involved in cell cycle transitions and are up-regulated in samples from patients with glioblastoma

**Figure 2:**
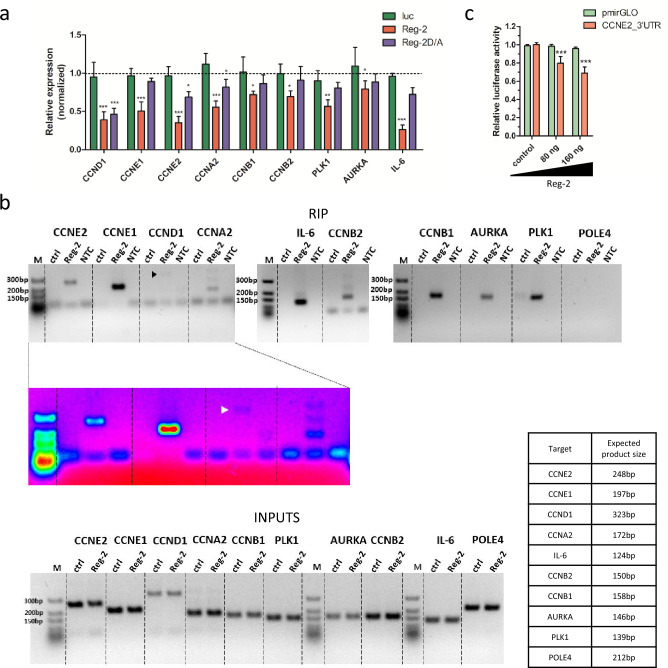
Reg-2 regulates the level of transcripts down-regulated in glioblastoma samples and involved in cell cycle regulation. (**a**) U87-MG cells with sleeping beauty (SB) transposon-based dox-inducible expression of luciferase (luc), wild-type Reg-2 (Reg-2), or mutein Reg-2 D196A (Reg-2D/A) were treated with dox (1 μg/ml) or left untreated for 24 h, and the levels of the indicated mRNAs were examined using qRT– PCR. Graphs show mean results presented as normalized values (induced/noninduced) from four independent experiments + / − SD. Data were analyzed using two-way ANOVA with Bonferroni's post-hoc test (*p < 0.05; **p < 0.01; ***p < 0.001). (**b**) Reg-2 directly binds endogenous mRNA encoding tested proteins. HeLa cells were transfected with vectors encoding the Reg-2-D/A mutein (with N-terminal HALO-Tag and TwinStrep Tag) or a luciferase-coding control vector, and after UV-crosslinking, the RNA immunoprecipitation (RIP) was performed. The immunoprecipitated RNA as well as RNA isolated from input samples were used for reverse transcription and qRT-PCR. Next, qRT-PCR products were run on 2% agarose gel with Ultra Low Range DNA Ladder (Thermo Fisher Scientific). In case of *CCND1*, increased contrast image with pseudocolor mask for visualization of low intensity bands is also presented. Image was prepared using ImageJ software. (**c**) Reg-2 is involved in the 3′UTR- dependent destabilization of *CCNE2* transcript. U251- MG cells were transfected with vectors encoding luciferase with the attached 3′UTR of *CCNE2* or without any additional 3′UTR (pmirGLO) and an expression vector for Reg-2 (80 ng or 160 ng). The graphs show the mean results of three independent experiments + /− SD. The data were analyzed using two-way ANOVA and Bonferroni's post hoc test (***p < 0.001).

To identify mRNA targets implicated in cell cycle regulation, we have performed an RNAseq analysis of the transcriptome of U251-MG human glioblastoma cells overexpressing Reg-2 or luciferase (control). Obtained results have been deposited in NCBI’s Gene Expression Omnibus and are accessible through GEO Series accession number GSE248634. Of the 29 analyzed transcripts encoding proteins involved in the regulation of proliferation, 21 mRNAs encoding cyclins, kinases, and regulatory proteins were significantly down-regulated by Reg-2 (Table 1). Since our results indicate the involvement of Reg-2 in cell cycle control during the G1/S transition, we have selected the *CCND1, CCNE1, CCNE2,* and *CCNA2* mRNAs (encoding cyclins D1, E1, E2, A2 respectively) for further analysis^12^. Interestingly, among transcripts affected by Reg-2, there are also mRNAs encoding proteins required for G2/M phases of the cell cycle^12^. To verify these data we have chosen *CCNB1* (encoding cyclin B1)*,* and *CCNB2* (encoding cyclin B2) for additional examination. We have also selected two additional transcripts encoding kinases important for cell cycle progression: *PLK1* encoding Polo-like kinase 1, a critical regulator of cell cycle progression, and *AURKA* encoding Aurora kinase A involved in different aspects of mitotic control. During cell cycle progression the functional cross-talk between these two kinases exists and both are important players in glioblastoma development^13,14^. Low levels of their mRNAs have been shown to have antiproliferative effects in glioma^15^. Results obtained by qPCR in U87-Reg-2 and U87-Reg-2D/A indicate that the levels of *CCNE1*, *CCNB1*, *CCNB2*, *PLK-1,* and *AURKA* are restricted by Reg-2 in an NYN/PIN-dependent manner. The levels of *CCND1, CCNE2,* and *CCNA2* are down-regulated by Reg-2 independently of the active nucleolytic domain (Fig. 2a). As a positive control in this analysis, we have used a well-known Reg-2 target, mRNA for IL-6. Consistent with previously published results, Reg-2 regulates *IL-6* mRNA in a NYN/PIN-dependent manner (Fig. 2a).

**Table 1:** Differential expression of selected mRNAs involved in proliferation and cell cycle regulation in Reg-2-overexpressing vs control U251-MG cells based on RNAseq analysis.

Category	Gene	Description	ENSEMBL IDENTIFIER	Reg-2 vs ctrl fold change	p-value	FDR p-value
CYCLINS	**CCNE2**	**Essential for the control of the cell cycle at the late G1 and early S phase**	**ENSG00000175305**	** − 2.11**	**0**	**0**
	**CCNE1**	**Essential for the control of the cell cycle at the G1/S transition**	**ENSG00000105173**	** − 1.43**	**6.19E–06**	**8.74E–05**
	**CCND1**	**Regulates the cell-cycle during G1/S transition**	**ENSG00000110092**	** − 1.87**	**0**	**0**
	CCND3	Regulates the cell-cycle during G1/S transition	ENSG00000112576	** − **1.31	1.61E–04	1.61E–03
	**CCNB1**	**Essential for the control of the cell cycle at the G2/M transition**	**ENSG00000134057**	** − 1.21**	**7.40E–03**	**0.05**
	**CCNB2**	**Essential for the control of the cell cycle at the G2/M transition**	**ENSG00000157456**	** − 1.21**	**7.12E–03**	**0.04**
	**CCNA2**	**Controls both the G1/S and the G2/M transition phases of the cell cycle**	**ENSG00000145386**	** − 1.26**	**9.99E–04**	**8.13–03**
	CCNG2	Negative regulation of cell cycle progression	ENSG00000138764	** − **1.3	4.36E–04	3.94E–03
KINASES	CDKN2D	Inhibition of CDK4 and CDK6	ENSG00000129355	** − **1.6	4.31E–10	1.26E–08
	CDK6	Serine/threonine kinase involved in the control of the cell cycle; promotes G1/S transition	ENSG00000105810	** − **1.48	1.88E–08	4.28E–07
	CDK2	Serine/threonine kinase involved in the control of the cell cycle; Acts at the G1/S transition and modulates G2 progression	ENSG00000123374	** − **1.42	1.60E–13	7.23E–12
	CDK11B	Plays multiple roles in cell cycle progression, cytokinesis and apoptosis	ENSG00000248333	** − **1.21	0.01	0.06
	AURKB	Serine/threonine kinase, component of the chromosomal passenger complex (CPC) that acts as a key regulator of mitosis	ENSG00000178999	** − **1.2	0.01	0.07
	**AURKA**	**Mitotic serine/threonine kinase that contributes to the regulation of cell cycle progression**	**ENSG00000087586**	** − 1.18**	**0.02**	**0.09**
	CDK7	Serine/threonine kinase involved in cell cycle control and in RNA polymerase II-mediated RNA transcription	ENSG00000134058	1.28	6.39E–04	5.53E–03
	CDK1	Catalytic subunit of the kinase complex known as M-phase promoting factor (MPF), which is essential for G1/S and G2/M phase transitions of cell cycle	ENSG00000185324	** − **1.03	0.66	0.82
	**PLK1**	**Critical regulator of cell cycle progression, mitosis, cytokinesis, and the DNA damage response**	**ENSG00000166851**	** − 1.37**	**1.81E–05**	**2.28E–04**
Cell division control proteins	CDC6	Participates in checkpoint controls that ensure DNA replication is completed before mitosis is initiated	ENSG00000094804	** − **1.66	1.24E–12	4.98E–11
	CDC14B	Dual-specificity phosphatase involved in DNA damage response. Essential regulator of the G2 DNA damage checkpoint	ENSG00000081377	** − **1.45	1.29E–06	2.12E–05
	CDC25A	Required for progression from G1 to the S phase of the cell cycle	ENSG00000164045	** − **1.4	3.04E–06	4.58E–05
	CDC20	Regulatory protein interacting with several other proteins at multiple points in the cell cycle	ENSG00000117399	** − **1.37	9.13E–06	1.24E–04
	CDC25B	Phosphatase that activates the cyclin dependent kinase CDC2; required for entry into mitosis	ENSG00000101224	** − **1.23	2.47E–05	3.02E–04
	CDC27	Component of the anaphase promoting complex/cyclosome (APC/C), that controls progression through mitosis and the G1 phase of the cell cycle	ENSG00000004897	** − **1.2	9.44E–03	0,056,301
	CDCA8	Component of the chromosomal passenger complex that is an essential regulator of mitosis and cell division	ENSG00000134690	** − **1.17	0,025,054	0,12,479
	CDC23	Essential for cell cycle progression through the G2/M transition	ENSG00000094880	** − **1.17	1.65E–03	0,012,551
	CDC45	Required for initiation of chromosomal DNA replication	ENSG00000093009	** − **1.11	0,138,836	0,415,178
	CDC14A	Dual-specificity phosphatase; required for centrosome separation and cytokinesis during cell division	ENSG00000079335	1.19	0,046,695	0,200,883
	CDC7	Kinase that phosphorylates critical substrates regulating the G1/S phase transition and initiation of DNA replication	ENSG00000097046	1.25	6.56E–06	9.19E–05
	CDC26	Component of the anaphase promoting complex/cyclosome (APC/C), that controls progression through mitosis and the G1 phase of the cell cycle	ENSG00000176386	1.3	2.66E–04	2.53E–03

Table shows fold change of selected transcripts together with p-value and FDR-adjusted p-value.

Transcripts selected for further analysis are bold.

To study the mechanisms of observed changes in transcript levels we have employed RNA immunoprecipitation (RIP). Cells were modified by SB transposon enabling the expression of Reg-2-D/A with N-terminal HALO-Tag and TwinStrep Tag. We have chosen Reg-2-D/A for these experiments to avoid target degradation. PCR analysis of immunoprecipitated mRNAs reveals direct binding of Reg-2 to all tested transcripts. As a positive control, we have used IL-6 mRNA and, as a negative control, POLE4 mRNA. Reg-2 is bound to the IL-6 transcript and does not interact with POLE4 mRNA (Fig. 2b).

To confirm that the observed regulation depends on the interaction of Reg-2 with the 3'UTRs of the transcripts, we have cloned the 3’UTR of *CCNE2* into a luciferase reporter vector. The co-expression of luciferase mRNA with attached *CCNE2* 3’UTR and the expression vector encoding Reg-2 results in decreased luciferase activity in transfected cells. Reg-2 overexpression correlates with a lower luciferase activity in a dose-dependent manner. The regulation requires the presence of *CCNE2* 3’UTR, as no decrease in luciferase activity is observed when this region is absent (Fig. 2c).

**Figure 3:**
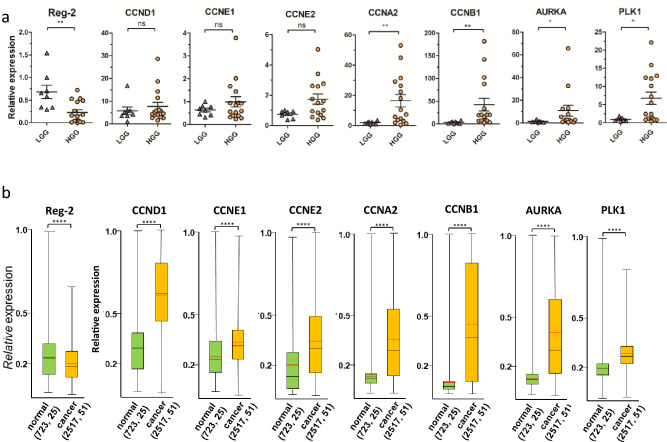
(**a**) The level of *Reg-2, CCND1*, *CCNE1*, *CCNE2*, *CCNA2*, *CCNB1*, *AURKA,* and *PLK1* in clinical samples of low-grade glioma (LGG) and glioblastoma multiforme (GBM). Tumor samples were homogenized and subjected to RNA isolation. Each point represents a single patient. The levels of the indicated mRNAs were examined using qRT–PCR. The horizontal lines represent the mean + / − SEM. The data were analyzed using the Mann–Whitney test. (*p < 0.05; **p < 0.01). (**b**) Expression of selected transcripts in normal and cancer tissues of the human brain. Numbers in parentheses indicate the number of samples and data sets, respectively. The red line indicates the average, the black line the median, the box upper side the Q3 percentile and the lower side the Q1 percentile, and the upper and lower error bars indicate the maximum and minimum respectively. Graph prepared from the data obtained from the Oncopression database.

We have analyzed the level of Reg-2 and its newly identified targets that encode the cyclins *CCND1, CCNE1, CCNE2, CCNB1, CCNA2,* and the kinases *PLK1* and *AURKA* in glioma tumors. The quantity of Reg-2 shows a correlation with the tumor grade, being at its lowest level in GBM. Conversely, higher mean expression levels for all investigated Reg-2 targets were noted in GBM samples compared to LGG samples. However, due to the high level of heterogeneity of GBM samples, statistical analysis of the examined sample group acknowledged significant changes only for *CCNB1, CCNA2, PLK1,* and *AURKA,* not for *CCND1, CCNE1*, and *CCNE2* (Fig. 3a). Nonetheless the data from publicly available databases confirm that the level of all investigated transcripts changes substantially between healthy and tumor tissue (Fig. 3b).

### Changes in Reg-2 targets are detected also on the protein level

**Figure 4:**
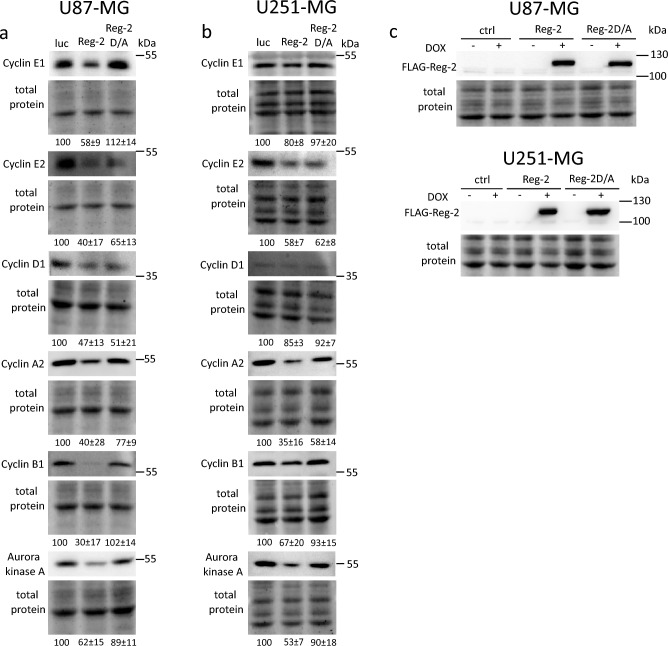
Reg-2 downregulates the level of proteins involved in cell cycle regulation. U87-MG or U251-MG with Sleeping Beauty (SB) transposon-based dox-inducible expression of luciferase (luc), wild-type Reg-2 (Reg-2) or mutein Reg-2 D196A (Reg-2 D/A) were treated with dox (1 μg/mL) for 24 h, then protein lysates were collected, and analyzed by western blotting. A representative image of two to three independent experiments with similar results is shown. Values below lanes show densitometry analysis (ratio of analyzed protein to total protein was normalized to control sample and presented as mean ± SD).

To confirm that the presence of Reg-2 correlates with decreased levels of proteins encoded by the newly discovered Reg-2 substrates, we have employed Western Blot analysis. In U87-Reg-2 cells overexpressing wild-type Reg-2 the reduction in the level of cyclin D1, E1, E2, A2, B1, and Aurora kinase A is observed. Of these proteins, cyclin D1, E2, and A2 are also decreased in U87-Reg-2D/A cells, confirming the NYN/PIN-independent mechanism of their regulation by Reg-2 (Fig. 4). We have performed a similar analysis in another glioblastoma cell line, U251-MG. The cells were modified using the SB system containing the coding sequence for Reg-2 (U251-Reg-2), Reg-2-D/A (U251-Reg-2D/A) or luciferase (control) under the dox-inducible promoter. The obtained results reveal a similar pattern of the regulation of the investigated proteins. Cyclins D1, E1, E2, B1, and Aurora kinase A are down-regulated in U251-Reg-2 but cyclins D1, E2, and A2 are also decreased in U251-Reg-2D/A cells. (Fig. 4). Obtained results show that changes in the amount of investigated proteins reflect changes detectable on the mRNA level.

## Discussion

All Reg/ZC3H12/MCPIP family members of RNases are involved in transcript turnover. Their specificity partly overlaps, for example, *IL-6* mRNA is degraded by all family members. However, it is hard to determine the extent of the overlapping substrates as our knowledge about this family's targets is mainly limited to identified Reg-1 targets. So far, merely a few Reg-2 substrates have been identified, namely *IL-6, IL-1, IER3*, and *Reg-1* mRNAs. In this report, we demonstrate that overexpression of Reg-2 in human glioblastoma cell lines down-regulates the level of several transcripts involved in the regulation of cell cycle progression such as *CCND1, CCNE1, CCNE2, CCNA2, CCNB1, CCNB2, PLK1,* and *AURKA*. By RIP we have confirmed that this regulation takes place through the direct interaction of Reg-2 with investigated transcripts. The amount of Reg-2 is reduced in samples from patients with GBM, thus the decrease in its level may be one of the mechanisms responsible for disturbances in cell cycle advancement. Abnormal activity of the cell cycle machinery is detected in all tumors and is the foundation of tumorigenesis. Well-documented changes in cell homeostasis linked to the control of DNA replication and mitosis include changes in gene expression, protein abundance, and protein post-translational modification. Recently, changes in transcript turnover have also been described as an important mechanism involved in the control of cell division; however, our knowledge about this aspect is very limited. So far, only two RNases participating in this process have been identified: ribonuclease mitochondrial RNA processing (MRP) and Reg-1. MRP is involved in cell cycle regulation and mitochondrial DNA replication^16^. This RNase cleaves *CLB2* mRNA in its 5’UTR, followed by its degradation by Xrn1 exonuclease. Disturbances in MRP activity caused by mutations lead to an increased level of *CLB2* and the encoded protein Clb2p (B-cyclin). As a result, late anaphase is delayed^17^. Mutation in the MRP subunit causes a recessively inherited developmental disorder, cartilage-hair hypoplasia^18^. Reg-1 overexpression restricts the level of *CCND1, CCND3, CCNE1, CCNE2, CDC2, AURKA,* and *MCM10* transcripts in the MV3 human melanoma cell line^19^. In human neuroblastoma cell lines, KELLY and BE(2)-C, Reg-1 down-regulates *CCND3, CCNE1, CCNE2, CCNA2,* and *CCNB1* mRNA^20^. In samples from patients with melanoma or neuroblastoma, the reduction of Reg-1 level has been detected, thus abnormalities in cell cycle progression in these tumors can result from the down-regulation of Reg-1^19,21^.

Glioblastoma cells that exhibit increased levels of Reg-2 undergo a reduction in their uncontrolled cell division potential. Elevated expression of Reg-2 results in the suppression of both their proliferation and their ability to form colonies (Fig. 1). This phenomenon is predominantly dependent on the RNase activity of Reg-2, as the overexpression of inactive/mutated form of Reg-2 only marginally impedes proliferation, without any noticeable effect on colony formation. Reg-2 overexpression stalls glioblastoma cells in the G1 phase, and among its newly identified targets we have detected mRNAs for cyclin D1, and cyclins E1 and E2 that act in the early and late G1/S phase respectively. However, Reg-2 also regulates the level of transcripts of cyclins that predominate in other cell cycle phases such as cyclin A2, B1, and B2 (phase G2/M). Furthermore, Reg-2 restricts the levels of *PLK1* and *AURKA* mRNAs encoding regulatory serine/threonine kinases involved in mitotic entry in an NYN/PIN-dependent manner. These kinases are in complex interplay through the cell cycle progression, with Aurora kinase A involved in the phosphorylation-mediated activation of PLK1^13^. Both kinases are overexpressed in glioma, and their high level is correlated with a worse prognosis in patients. Both are also considered to be potential targets for glioblastoma therapy^22,23^. Down-regulation of their mRNA level is correlated with an antiproliferative effect^15^. Some of the identified Reg-2 targets, *CCND1, CCNE2,* and *CCNA2* are down-regulated independently of the presence of its active endonucleolytic domain, although the effect of wild-type Reg-2 is more profound than that of inactive mutein. The impact of mutein on the transcript level suggests a possible role of Reg-2 in the recruitment of other nucleases to Reg-2 targets. So far, the interaction of Reg-1 and Reg-4 on *IL-6* mRNA has been described^24^. Hence, a plausible explanation for the observed impact of Reg-2D/A on the levels of CCND1, CCNE2, and CCNA2 mRNAs could involve the interaction of Reg-2 with other enzymes acting on these transcripts. The proteins from the Reg/MCPIP/ZC3H12 family recognize stem-loop structures located in the 3’UTR of their targets^6,25^. We have chosen one of the newly identified Reg-2 substrates, *CCNE2,* to study this issue. *CCNE2* 3’UTR cloned into the reporter vector changes the stability of reporter mRNA. Reg-2 overexpression negatively regulates the level of the reporter protein translated on the mRNA containing *CCNE2* 3’UTR. This regulation is dose-dependent.

In this paper, we emphasize the existence of a novel way of regulating the cell cycle. The RNases involved in transcript turnover accompaniment well-known elements such as transcription factors regulating the expression of genes encoding proteins engaged in the cell cycle and machinery responsible for their posttranslational modifications and degradation.

Our results indicate a key role of Reg-2 in glioblastoma biology. Through the regulation of neuroinflammation and restriction of proliferation, Reg-2 controls crucial elements of glioblastoma development.

## Materials and methods

### Cell culture

The U251-MG (ECACC 09063001), and U87-MG (ECACC 89081402) cell lines (wild type and modified) were cultured in Dulbecco’s modified Eagle’s medium (DMEM) with 4.5 g/L glucose (Biowest) supplemented with 10% fetal bovine serum without tetracycline (Biowest). The HeLa cell line was cultured in Dulbecco's modified Eagle’s medium (DMEM) with 1.0 g/L d-glucose (Lonza) supplemented with 2 mM l-glutamine (Sigma-Aldrich) and 10% fetal bovine serum (Biowest). Cell lines modified with the Sleeping Beauty transposon system were additionally supplemented with 1 µg/ml puromycin (Invitrogen). Cell lines were maintained at 37 °C in a humidified atmosphere with 5% CO_2_. Cells were regularly examined for mycoplasma contamination using PCR^26^.

### Establishment of cell lines with inducible expression of Regnase-2

U251-MG or U87-MG cells were seeded in a 12-well plate and the following day the cells were transfected using Lipofectamine 3000 (Thermo Fisher Scientific) with 900 ng of pSBtet-GP, pSBtet-GP-Regnase-2, pSBtet-GP-Regnase-2-D/A, and 100 ng of the pCMV(CAT)T7-SB100 vector. 24 h later cells were trypsinized and one-fifth was transferred into 6-well plates. Cells transfected with transposon vectors were selected using puromycin (1 µg/ml, Invivogen) for seven days. pCMV (CAT)T7-SB100 was a gift from Zsuzsanna Izsvak (Addgene plasmid #34879^27^, pSBtet-GP was a gift from Eric Kowarz (Addgene plasmid #60495)^28^.

### Plasmid constructions

The construct pmirGLO-CCNE2_3'UTR that encodes the firefly luciferase transcript fused with the 3’UTR fragment of *CCNE2* was prepared by insertion of 3′UTR amplified by PCR amplified *CCNE2* 3′UTR (NM_057749.3) into the pmirGLO vector (Promega, USA). Briefly, the 3′UTR fragment of *CCNE2* was amplified from cDNA prepared from U251-MG cells using Q5 high-fidelity DNA polymerase (New England Biolabs, USA), and the following primers: forward 5′- GGTTTAAACAGAAGATAACTAAGCAAACAAGTTGG-3′ and back 5′- ATCTAGACAATCCAGAAGAAGTGTTACAGCT-3′. After agarose gel electrophoresis, the PCR product corresponding to the *CCNE2* 3′UTR fragment was excited, purified, cleaved with PmeI and XbaI (NEB, USA), and inserted using T4 DNA ligase (NEB, USA) into gel-purified pmirGLO vector linearized with PmeI and XbaI and dephosphorylated with CIP (NEB, USA). The construct was verified by Sanger sequencing (Genomed, Poland). The pSBtet-Pur-HALO-TST-Reg-2 vector was generated using NEBulider HiFi DNA Assembly Cloning Kit (New England Biolabs, USA) according to the manufacturer’s instructions. Fragments used for construct preparation were amplified using Q5 High Fidelity DNA Polymerase (New England Biolabs, USA). HRV 3C site and TwinStrep Tag fragment were amplified using the following primers: 5′-GCGGGGCGCGCCCTGGAAGTTCTGTTTCAGGGTCCG-3′ and 5′-CaCCtCCGGAtCCaCCAGCTTTCTCAAACTGAGGGTGGC-3′ with pET-HALO-KHNYN as a template. HALO-Tag fragment was amplified using the following primers: 5′-CCTGCAGGCACCATGGTGgcagaaatcggtactggctttcc-3′ and 5′-AACTTCCAGGGCGCGCCCcgcgccggaaatctcgagcgtc-3′ and backbone was amplified using following primers: 5′-tgtcaggccaagcttccatcg-3′ and 5′-caccatggtgcctgcagggg-3′ with pSBtet-HaloTag-Reg-2 as a template. The PSBtetPur-HALO-TST-Reg-2D/A vector was generated using Q5 Site-Directed Mutagenesis Kit (New England Biolabs) according to the manufacturer’s protocol using pSBtet-Pur-HALO-TST-Reg-2 vector as a template and following primers: 5′-CTGGAAGTAATGTGGCAATGAGCC-3′ and 5′- CAATGACAACTGGCCTCAAATTGTC-3′. The construction of SB transposon vectors used for inducible overexpression of Reg2/Reg2 D/A was previously described.

### Transfection, stimulation, and reporter gene assay

U251-MG cells were seeded in 24-well plates at a density of 5 × 10^4^ cells/well. The following day, the cells were transfected with PEI reagent (Polysciences, USA) according to the manufacturer’s instructions. A total amount of 800 ng of plasmid DNA per well was used, including 400 ng of the luciferase-coding reporter plasmid (pmirGLO or pmirGLO-CCNE2-3′UTR) and varying amounts of Reg-2 expression vector. The quantity of DNA/well was normalized using an empty pcDNA3.1/mycHisA vector (Invitrogen, USA). 24 h after transfection cells were lysed and firefly and *Renilla* luciferase activity were measured using Dual-Luciferase Reporter Assay System (Promega, USA), according to the manufacturer’s instructions. For transfection efficiency normalization the *Renilla* luciferase, encoded on the same plasmid as the firefly luciferase (pmirGLO), was used.

### RNA immunoprecipitation

HeLa cells with Dox-inducible expression of Reg-2D/A N-terminally tagged with HALO and TwinStrep Tags separated by HRV 3C cleavage site or luciferase (control cells, transfected with pSB-tet-Pur plasmid) were seeded on 150-mm cell culture dishes and the following day transgene expression was induced with 1 µg/ml Dox. After 24 h the cells were UV-irradiated at 254 nm with 400 mJ/cm^2^, lysed in modified RIPA buffer (50 mM HEPES pH 7.5, 150 mM NaCl, 1% Triton-X-100, 0.25% sodium deoxycholate, 3 mM MgCl_2_, 5 mM DTT, 1 × Protease Inhibitor Cocktail (Promega) and 1U/ml RNase Inhibitor (A&A Biotechnology) and homogenized using a syringe and a 23G needle. After homogenization cell debris was removed by centrifugation, 1/10 of the supernatants were kept as input samples, and the rest was used for two-step purification. In the first step the lysate was diluted 5 × in HaloTag® protein purification buffer (50 mM HEPES pH 7.5, 150 mM NaCl, 1 mM DTT, 0.005% IGEPAL® CA-630) supplemented with 1 × Protease Inhibitor Cocktail (Promega) and 0.2 U/ml RNase Inhibitor (A&A Biotechnology), and Reg-2-mRNA complexes were bound overnight at 4 °C to Magne® HaloTag® Beads (Promega). The bound complexes were washed 4 × with HaloTag® protein purification buffer and 3 × with HRV 3C Reaction buffer (Pierce). After washing the complexes were cleaved off the beads with HRV 3C Protease (Pierce) in HRV 3C Reaction buffer (Pierce) supplemented with 1U/ml RNase Inhibitor (A&A biotechnology) by overnight incubation at 4 °C. The following day the cleaved-off complexes were subjected to second-step purification. Briefly, the cleavage reaction was incubated with MagStrep "type3" XT beads (IBA Lifesciences) for 2 h at 4 °C, washed 4 × with 1 × Buffer W (IBA Lifesciences) with 6 M guanidium hydrochloride, 2 × with 1 × Buffer W, and eluted 3 × with 1 × Buffer BXT (IBA Lifesciences) containing 200 mM d-biotin. The pooled eluates were mixed 1:1 with 2 × proteinase K digestion buffer (20 mM Tris–HCl pH 8.0, 200 mM NaCl, 2 mM EDTA, 1% SDS) and digested for 0.5 h at 50 °C with 400 µmg/ml proteinase K (Ambion) along with the input samples. After digestion the RNA was purified by phenol/chloroform extraction and isopropanol precipitation with Pellet Paint NF (Merck) used as a coprecipitant. The purified RNA was reverse transcribed to cDNA using MMLV Reverse transcriptase (Promega) and a 1:1 mix of oligo(dT)_15_ and random hexamer primers and subjected to semiquantitative PCR for evaluation of the Reg-2 bound transcripts.

### Western blot analysis

Cells modified with SB transposons were seeded in 12-well plates. 24 h after transfection, protein expression was induced by adding doxycycline (1 µg/ml). The following day, cells were lysed in SDS loading buffer (0.35 M Tris·HCl, 35% (v/v) glycerol, 10% (w/v) SDS, 3.6 M β-mercaptoethanol, 0.12 g/ml bromophenol blue) and denatured at 95 °C for 7 min. Protein extracts were separated on SDS-PAGE electrophoresis, transferred to low fluorescence PVDF membrane (Millipore), and analyzed by Western blotting. For total protein UV-visualization 2,2,2-trichloroethanol (TCE) was added to polyacrylamide gels to a final concentration of 0.5% v/v^29^. The following antibodies were used: anti-CCNE1 (#4129, Cell Signaling Technology), anti-CCNE2 (#4132, Cell Signaling Technology), anti-CCNB1 (#4138, Cell Signaling Technology), anti-CCNA2 (#4656, Cell Signaling Technology), anti-cyclin D1 (#2978, Cell Signaling Technology), anti-Aurora kinase A (#14475, Cell Signaling Technology), monoclonal anti-FLAG M2 antibody produced in mice (F3165, Merck), anti-Lamin B1 (D9V6H, Cell Signaling Technology), anti-mouse-HRP (#7076) and anti-rabbit-HRP (#7074) (Cell Signaling). The blots were cut prior to hybridization with antibodies. Luminescence was detected using Clarity Western ECL Substrate (Bio-Rad) and recorded using the Fusion-Fx documentation system (Vilber Lourmat). Images were cropped and prepared using Quantity One software. All modifications (brightness and contrast adjustments) were applied equally across the entire image and applied equally to controls. Figures containing Western Blot images were prepared using Inkscape software. Original uncropped images with visible edges, as well as images combined with protein ladder are provided in Supplementary Informations. The densitometry analysis was performed on raw images using ImageJ software.

### RNAseq

To identify new targets of Reg-2 we performed RNAseq analysis. Using the SB transposon system we have modified U251-MG cells to generate a cell line with Dox-inducible expression of Reg2, or luciferase (control cells). To identify differentially expressed genes we extracted total RNA from control and Reg-2 overexpressing cells (using Zymo Research Direct-zol RNA MiniPrep kit, cat. R2053) and sent samples (in tryplicates) for complete analysis. Assessment of RNA quality, library preparation, RNA seq, and all data analysis was performed by Qiagen Genomic Services, Germany.

### Cell count/clonogenic assay

For clonogenic assay cells were seeded in triplicates at a density of 100 cells per well on 6-well plates. Doxycycline (1 µg/ml) was added daily to half of the wells to induce the expression of the transgene. When visible colonies appeared (after ~ 10 days) cells were fixed using a Clonogenic assay fixer/stain (6% vol/vol glutaraldehyde, 0.5% wt/vol crystal violet), and whole plates were scanned using the Olympus IX83 microscope. Quantitative analysis was performed by determining the percentage of area covered by crystal violet stained colonies using ImageJ software.

For cell count assay U87-MG-Reg-2/Reg-2 D/A and control (transfected with luciferase-coding vector) cells were seeded in 6-well plates at the density of 80,000 cells per well. To induce transgene expression, doxycycline (1 µg/ml) was added every 24 h. Cells were trypsinized and counted after 24, 48, and 72 h from expression induction using the TC-20 cell counter (BioRad).

### RNA isolation and reverse transcription

Tissue sample collection was performed following Jagiellonian University Ethics Committee guidelines and regulations. Informed consent was obtained from all subjects and/or their legal guardian(s). Before RNA isolation tissue samples were preserved in Nucleic Acid Preservation buffer (prepared according to M. Camacho-Sanchez et. al. 2013)^30^ and stored at − 80 °C. The samples were then homogenized in phenozol [1:1 phenol: GTC (4 M guanidinium thiocyanate, 25 nM sodium citrate, pH 7.0, 0.05% (w/v) sarkosyl and 0.1 M 2-mercaptoethanol)], and total RNA isolation was performed according to the Chomczynski modified protocol. Cells were lysed in GTC buffer and RNA isolation was performed according to Chomczynski’s protocol. RNA quantification and purity were assessed using spectrophotometric measurements using a NanoDrop ND-1000 spectrophotometer (Thermo Scientific). RNA integrity was assessed by denaturing, formaldehyde gel electrophoresis. The reverse transcription reaction was carried out using 1 µg of total RNA with M-MLV-Reverse transcriptase (Promega) and 500 ng of random hexamer primers (EurX) according to the manufacturer’s instructions. The cDNA was used in quantitative real-time PCR (qRT-PCR) for the evaluation of the amounts of the mRNAs of interest.

### qRT-PCR

qRT-PCR was performed using RT-HS-PCR-Mix-SYBR-A (A&A Biotechnology). The levels of mRNAs of interest in each sample were analyzed in duplicate and the expression level was normalized to *POLE4* or *TBP*. Expression levels were analyzed by the ΔΔCt method. The following primers were used: IL-6-for (GACAGCCACTCACCTCTTCA), IL-6-rev (AGTGCCTCTTTGCTGCTTTC), TBP-for (GCCAGCTTCGGAGAGTTCTGGGATT), TBP-rev (CGGGCACGAAGTGCAATGGTCTTTA), POLE4-for (TCAGAGGAGAGACTTGGATAATGC), POLE4-rev (GCTTATCCCGGCATAGGTGA), CCNE2-for (GCTGGTCTGGCGAGGTTTT), CCNE2-rev (AATGCAAGGACTGATCCCCC), CCNE1-for (CAGATGGAGCTTGTTCAGGAGAT), CCNE1-rev (TCAGCCAGGACACAATAGTCA), CCNA2-for (AATGGATGGTAGTTTTGAGTCACCA), CCNA2-rev (TGGCTGTTTCTTCATGTAACCCA), CCNB2-for (AGCACAAGTAGCTAAGAAAGCTC), CCNB2-rev (TCAGGTGTGGGAGAAGGACC), CCNB1-for (TGTTGGTTTCTGCTGGGTGT-3), CCNB1-rev (TGCCATGTTGATCTTCGCCT) PLK1-for (TCAATGCCTCCAAGCCCTC), PLK1-rev (TTATCACAGAGCTGATACCCAAG), AURKA-for (GTCAAGTCCCCTGTCGGTTC), AURKA-rev (CGGTCCATGATGCCTCTAGC), CCND1-for (CCGCTGGCCATGAACTACCT), CCND1-rev (ACGAAGGTCTGCGCGTGTT)^20,31^.

### Cell proliferation assay

Cell proliferation was examined using the CellTrace Far Red Cell Proliferation Kit (Cat. No. C34564, Invitrogen) according to the manufacturer’s instructions. Briefly U87-MG Reg-2/Reg-2D/A, or control (transfected with luciferase-encoding vector) cells were stained with 2.5 µM CellTrace Far Red reagent, split into two dishes, and one of the dishes was treated with 1 µg/mL doxycycline for induction of recombinant protein expression. After 48 h of induction, the cells (50,000 FSC/SSC gated cells/sample of induced and non-induced cells) were analyzed for CellTrace Far Red fluorescence intensity by flow cytometry using the BD FACSCalibur cytometer (Becton Dickinson). The BD CellQuest Pro software (Becton Dickinson) was used for data acquisition and FlowJo (v10.4.2, FlowJo) was used for data analysis.

### Cell cycle analysis

U87-MG-Reg-2/Reg-2 D/A or control (transfected with luciferase-encoding vector) cells were treated with 1 μg/ml doxycycline for induction of recombinant protein expression for 48 h. Then, the cells were trypsinized, washed with PBS two times, resuspended in 2 mL of PBS, and fixed by dropwise addition of 6 mL of ice-cold 96% ethanol. After fixation, the cells were washed two times with PBS and stained with PI/SAP solution (0.05 mg/mL propidium iodide, 1 mg/mL RNase A, 0.02% saponin, 0.0001% Triton-X-100 in PBS) by incubation for 15 min at 37 °C. Immediately after staining, the cells (250,000 FSC/SSC gated cells/sample) were analyzed for propidium iodide fluorescence intensity by flow cytometry using the BD FACSCalibur cytometer (Becton Dickinson). BD CellQuest Pro Software (Becton Dickinson) was used for data acquisition. The percentage of cells in each cycle was calculated with the FlowJo (v10.4.2, FlowJo) Cell Cycle analysis platform.

### Statistical analysis

Statistical analysis was performed using Graph Pad Prism software (version 5.01, GraphPad Software Inc.). If not specified differently the asterisks denote the statistical significance of the indicated data point versus the control sample. The exact tests performed are indicated in the figure descriptions.

### Ethical approval and consent to participate

Jagiellonian University Ethics Committee approval for human glioblastoma samples collection and analysis #1072.6120.65.2020.

### Supplementary Information

Supplementary Information 1.

## Data Availability

Sequencing data have been deposited in NCBI's Gene Expression Omnibus and are accessible through GEO Series accession number GSE248634. Any additional data are available from the corresponding author upon reasonable request. All data supporting the conclusions of this article are included within the article. Information regarding the experimental methods used, and the data in this paper are available to scientific communities upon direct contact with the corresponding author (Aneta Kasza, email: aneta.kasza@uj.edu.pl).
