# Supplementary material for: Regnase-2 inhibits glioblastoma cell proliferation
Source: Sci Rep. 2024 Jan 18;14:1574. doi: 10.1038/s41598-024-51809-x (PMC10796923; doi:10.1038/s41598-024-51809-x)

Fig. 1a

FLAG and LaminB1 – original image

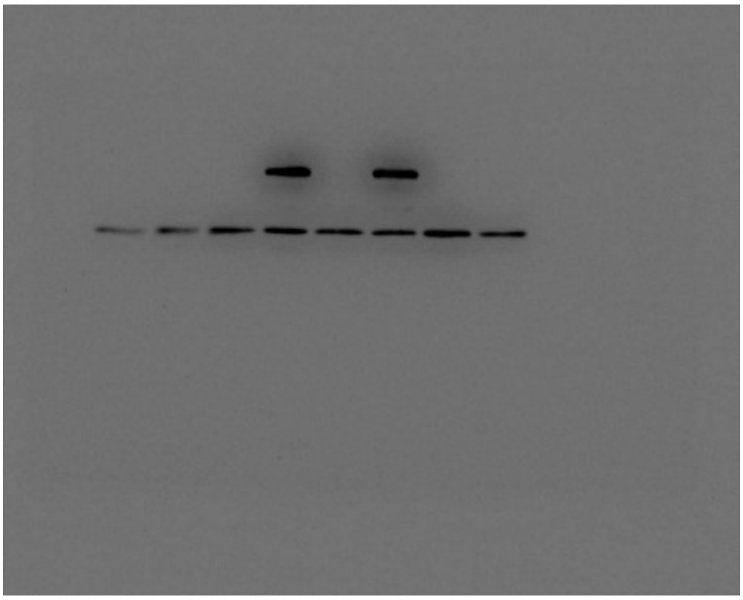

FLAG and LaminB1 – different exposure time

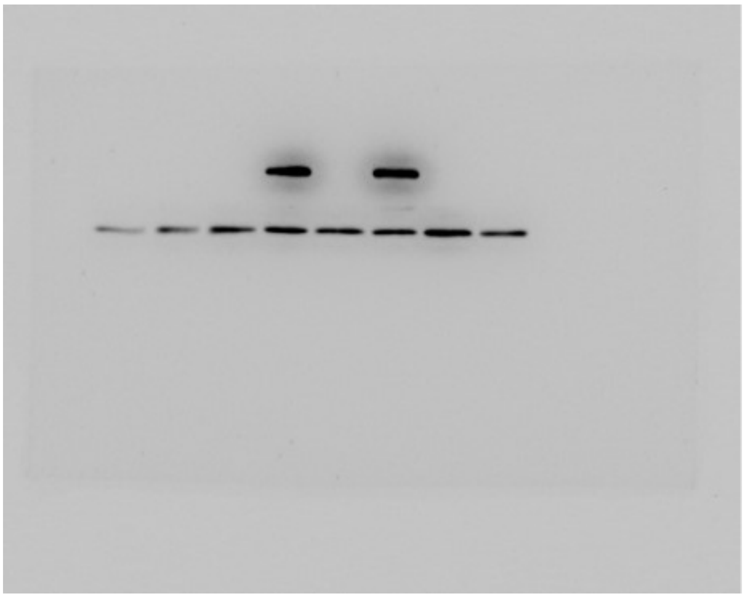

Oversaturated image with visible borders

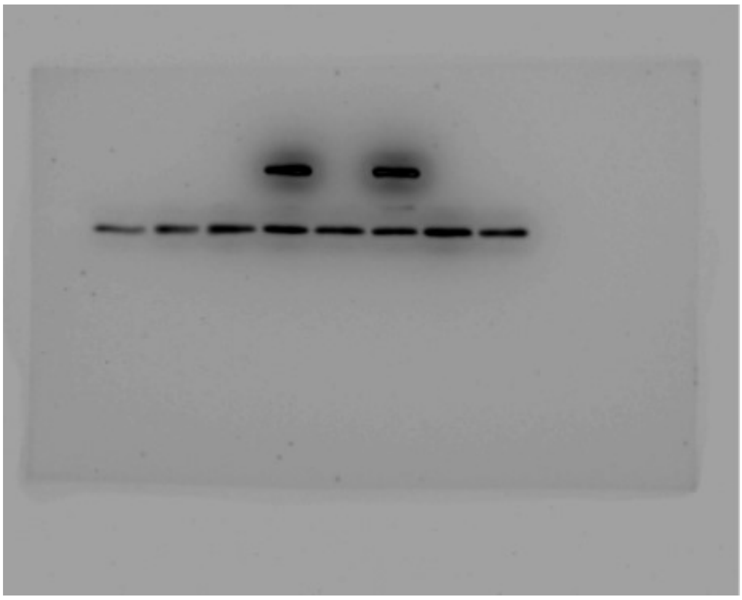

Image with protein ladder

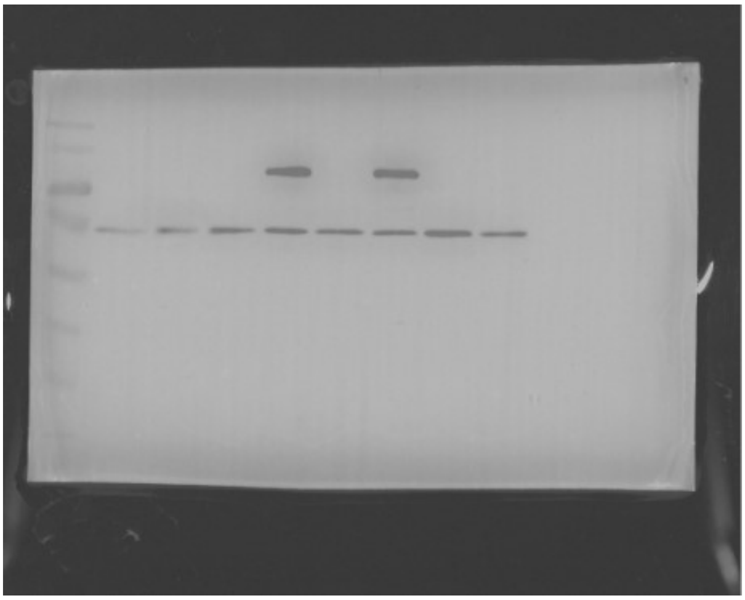

PageRuler Prestained Protein Ladder 10 to 180 kDa was used (ThermoFisher Scientific, cat. 26616)

Fig. 2b

RIP

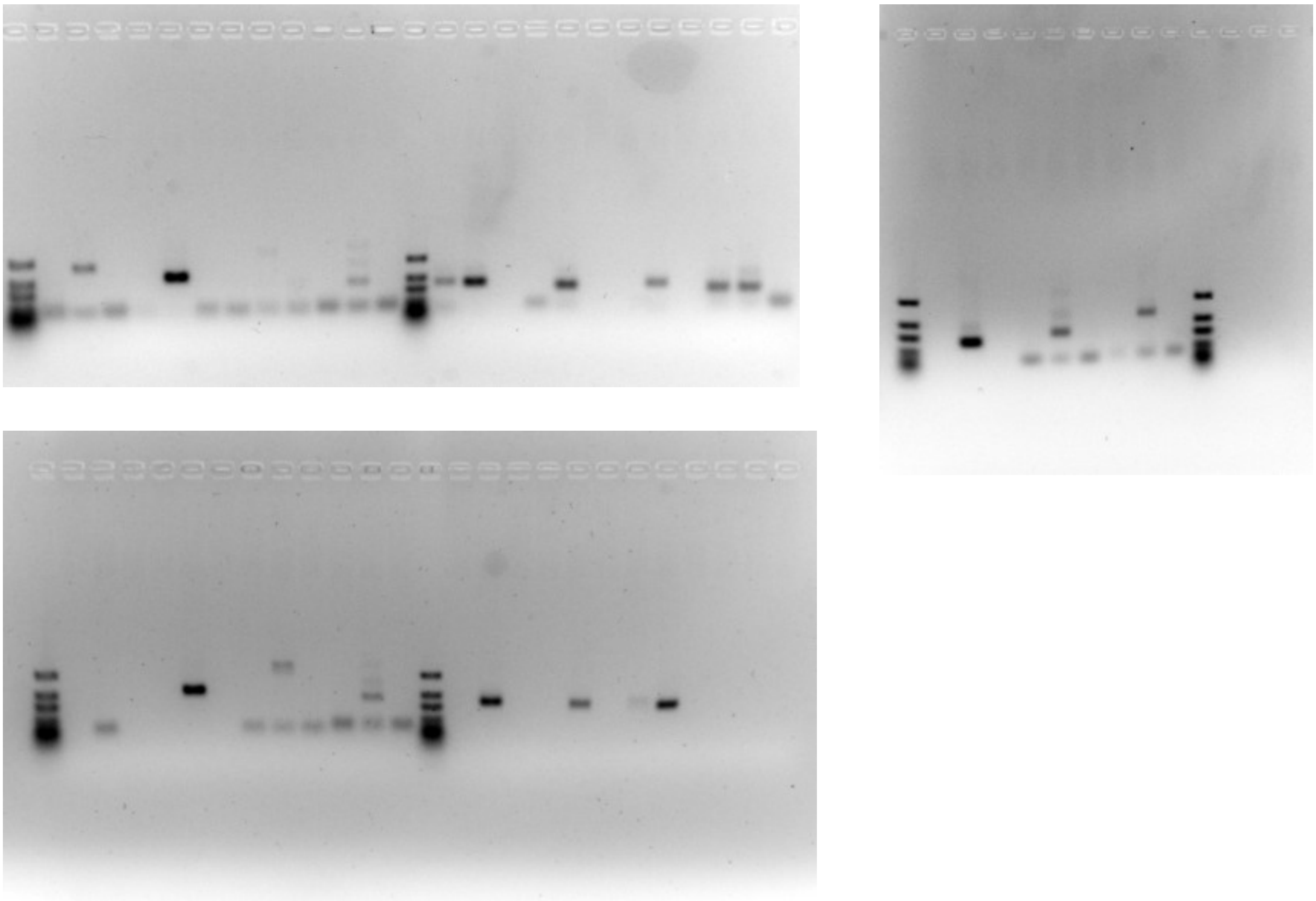

INPUTS

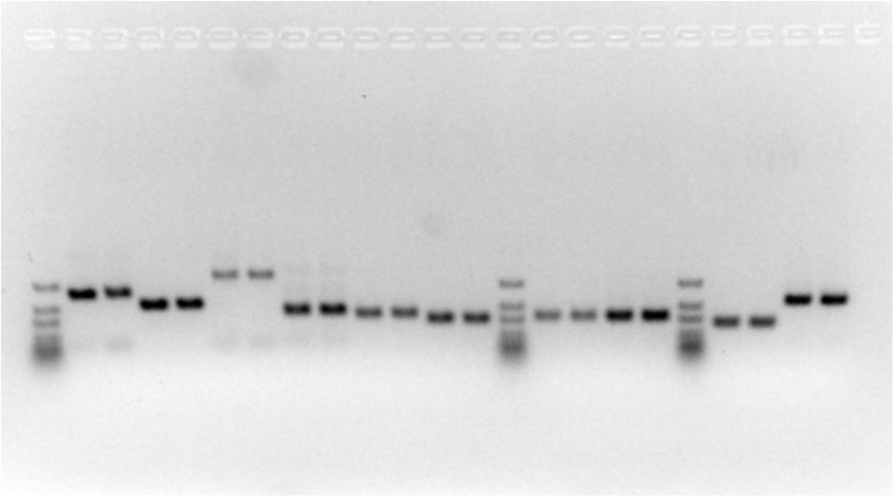

Fig. 4a

# U87-MG – original uncropped images

Cyclin E1

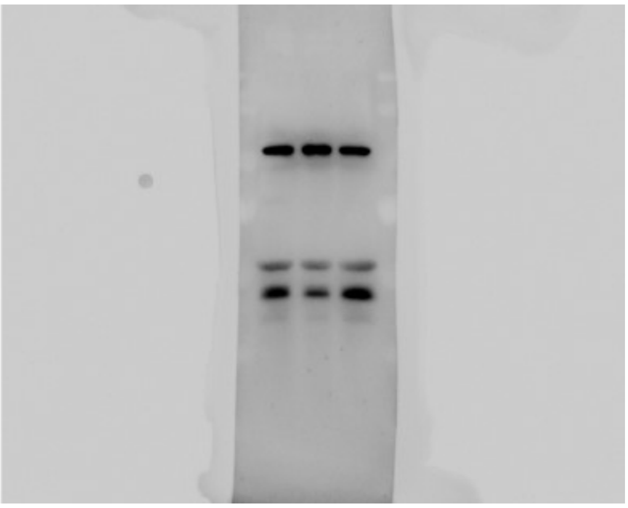

Cyclin E2

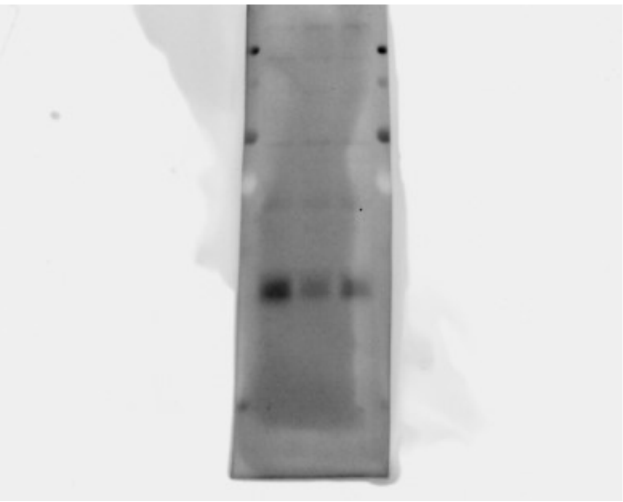

Cyclin D1

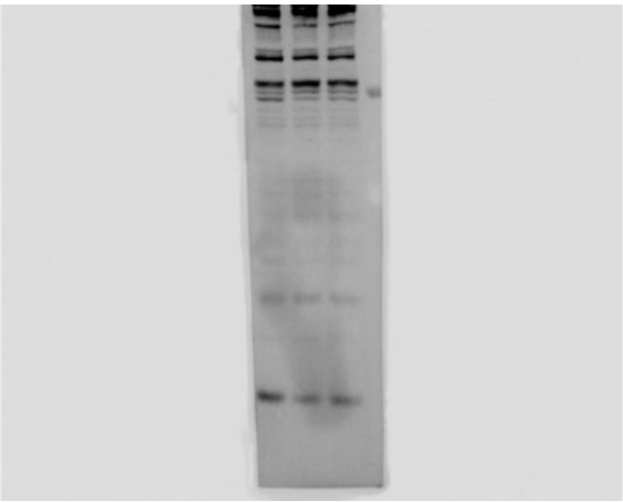

Cyclin A2

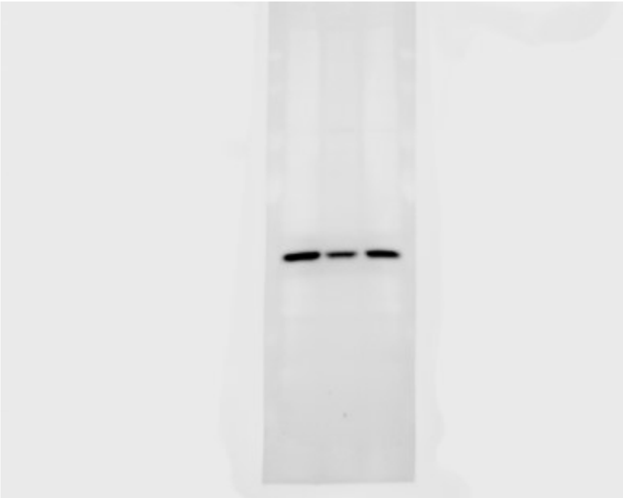

Cyclin B1

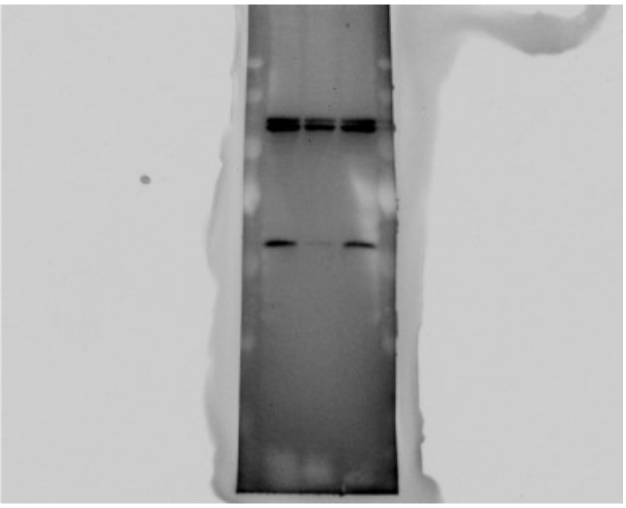

Aurora A kinase

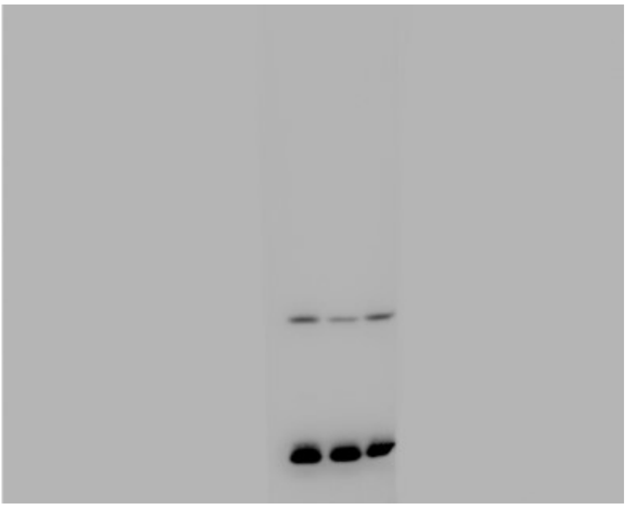

Fig. 4a

U87-MG – images with protein ladder

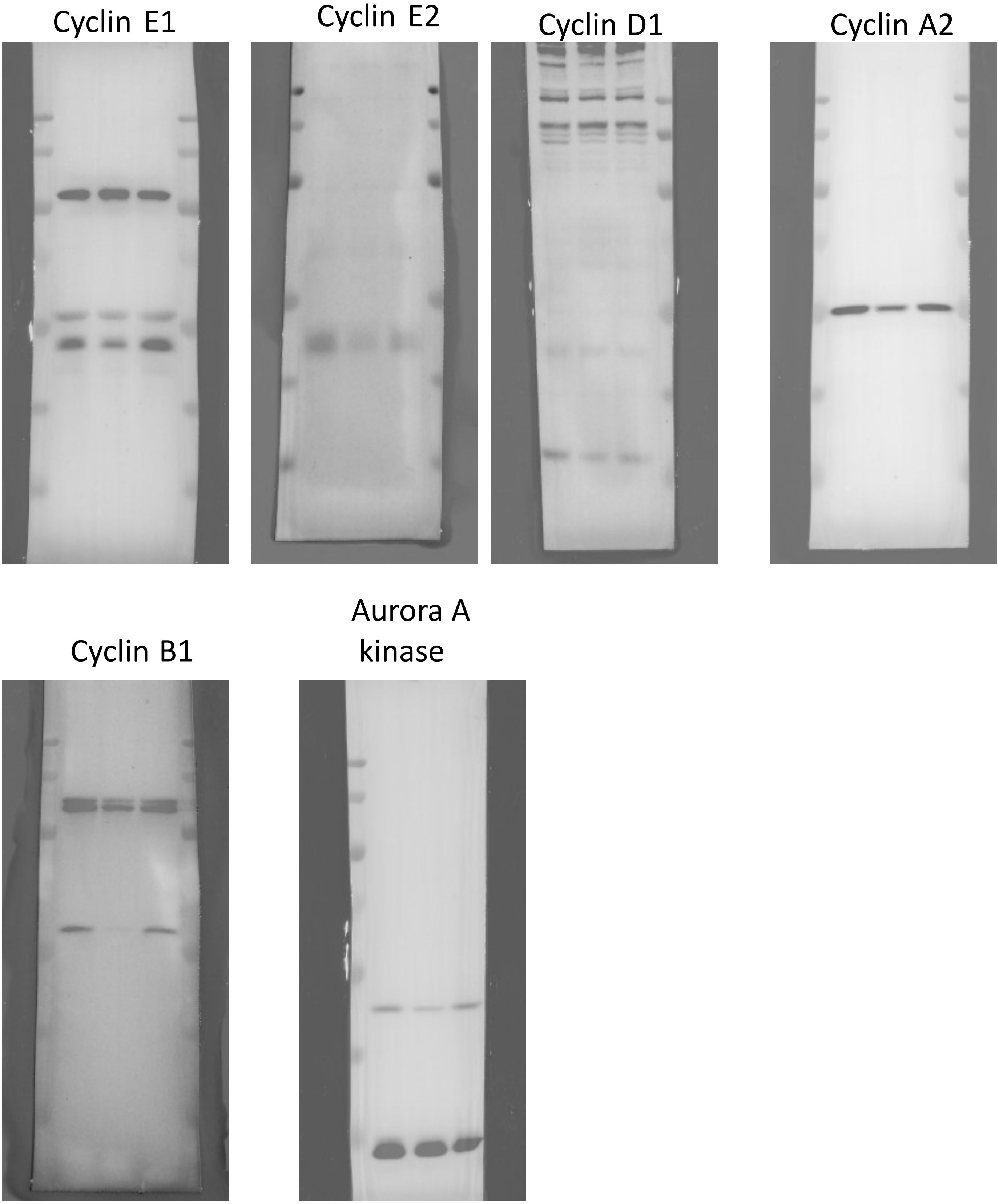


PageRuler Prestained Protein Ladder 10 to 180 kDa
was used (ThermoFisher Scientific, cat. 26616)

Fig. 4a

U87-MG cropped and adjusted

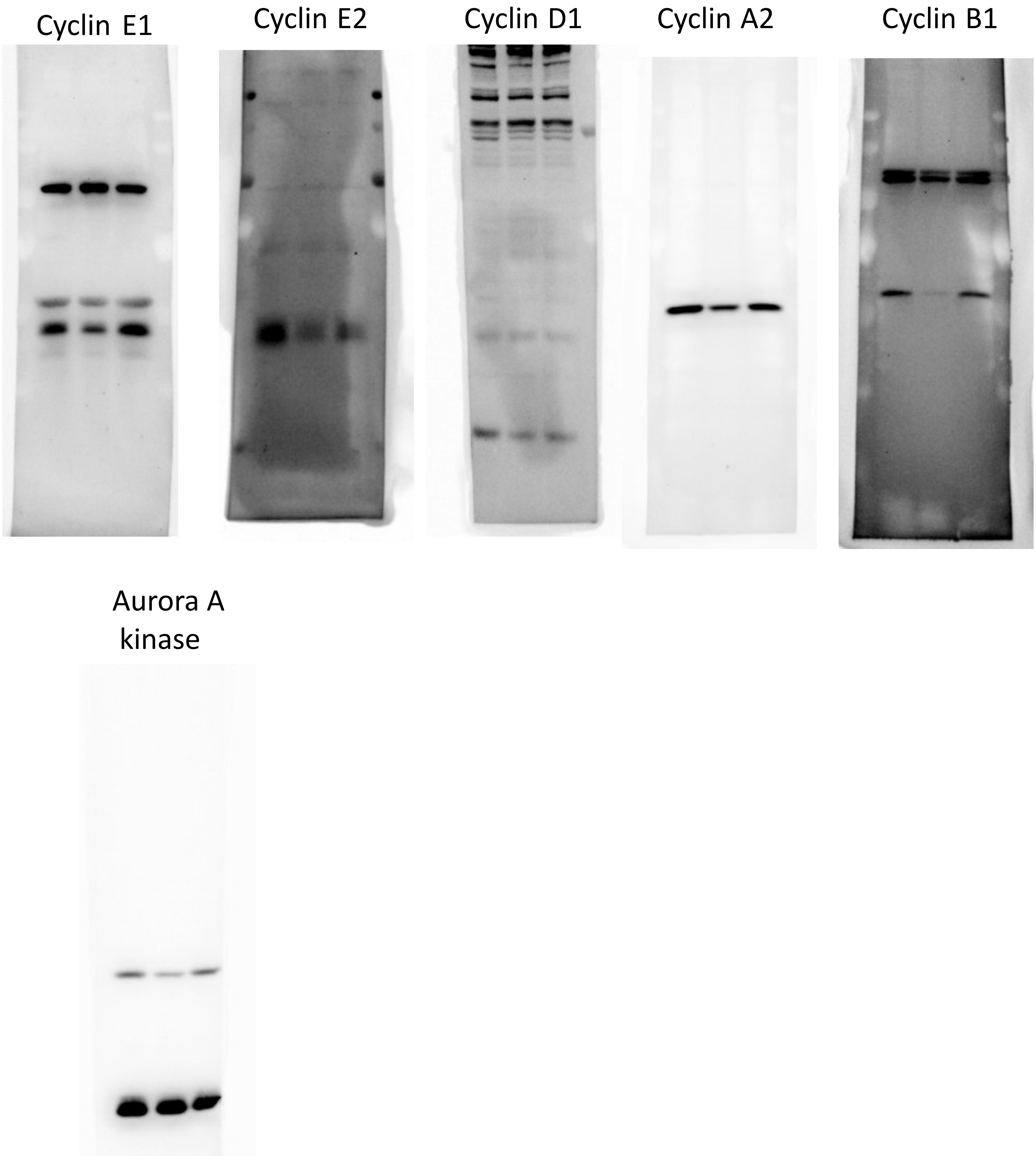

Fig. 4a

# U87-MG – Total protein

Cyclin E2 Cyclin E1

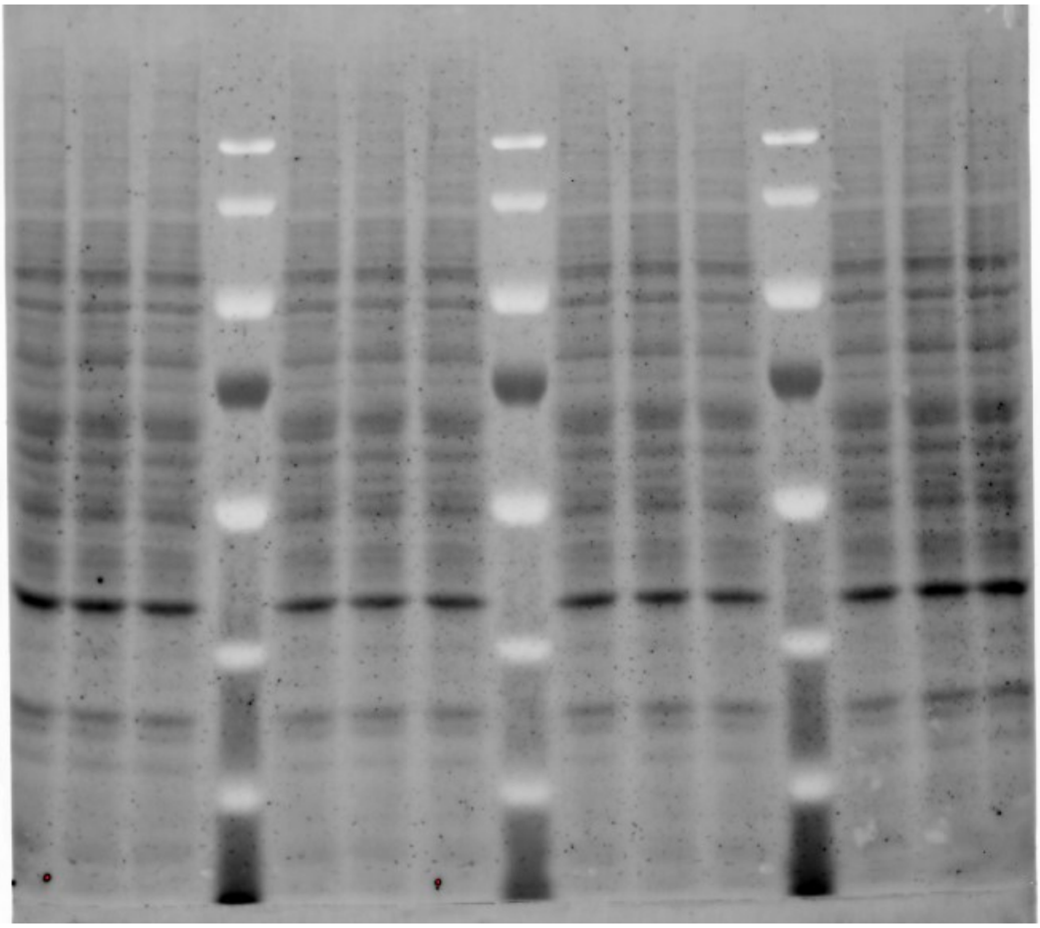

Cyclin D1 Cyclin A2 Cyclin B1 Aurora A kinase

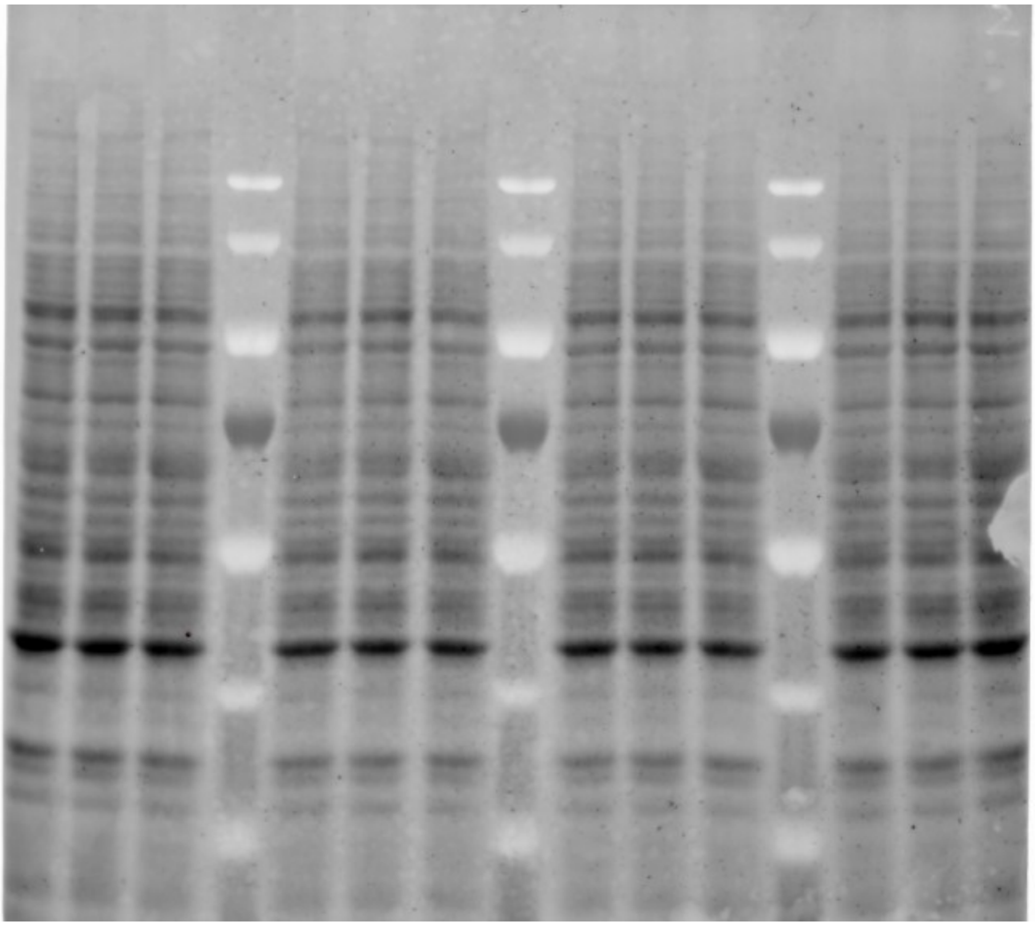

Fig. 4b

# U251-MG – original uncropped images

Cyclin E1

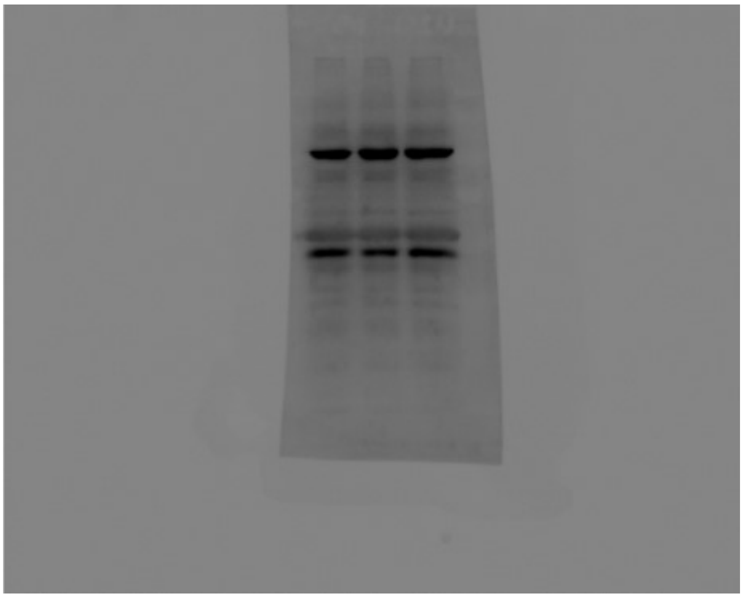

Cyclin E2

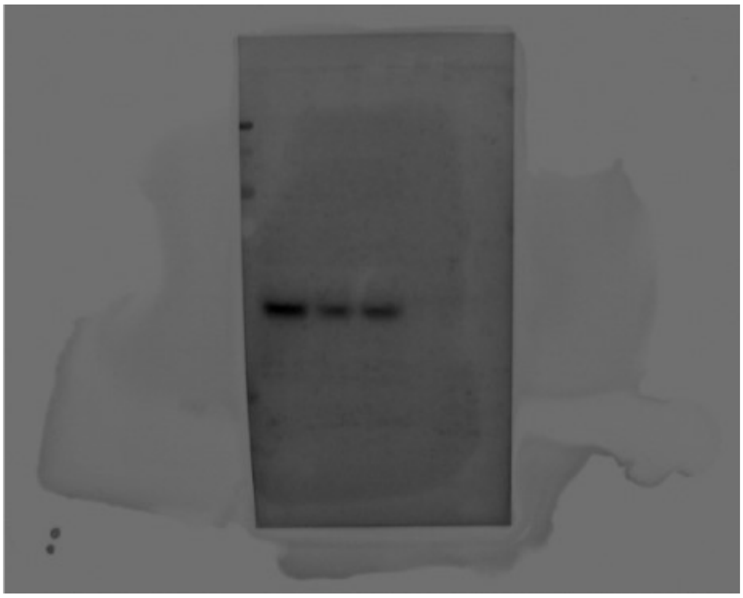

Cyclin D1

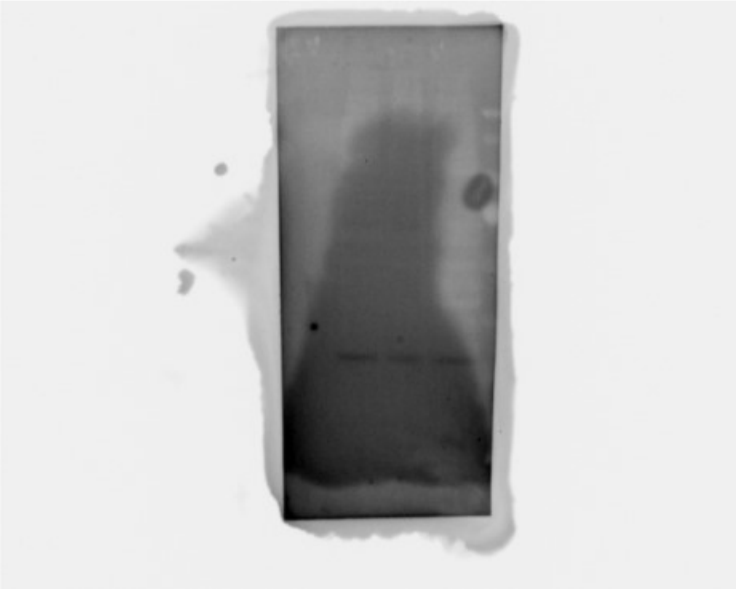

Cyclin A2

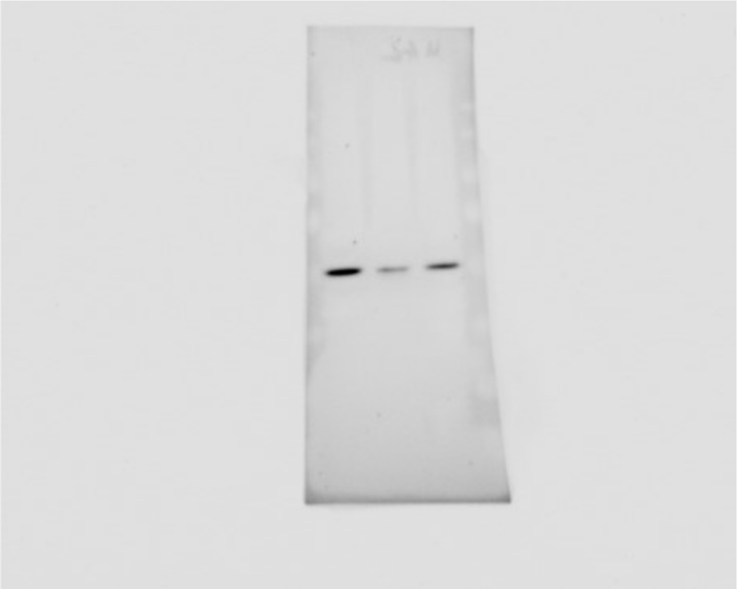

Cyclin B1

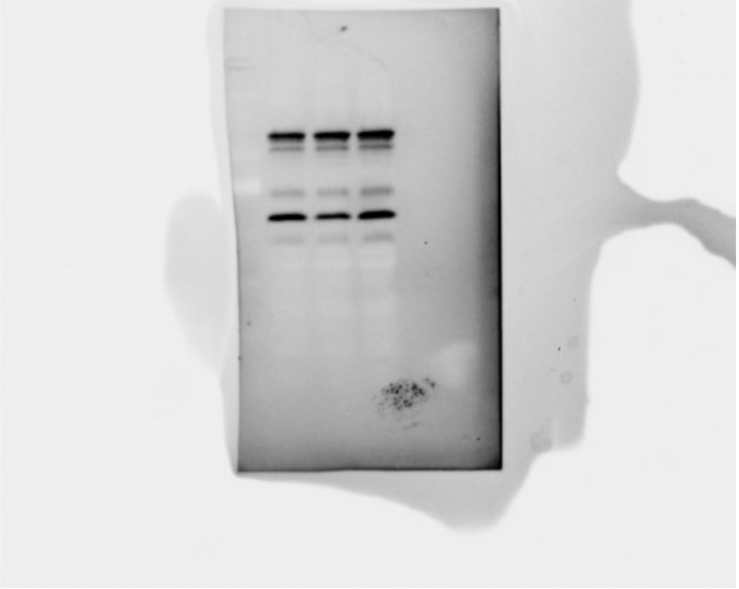

Aurora A kinase

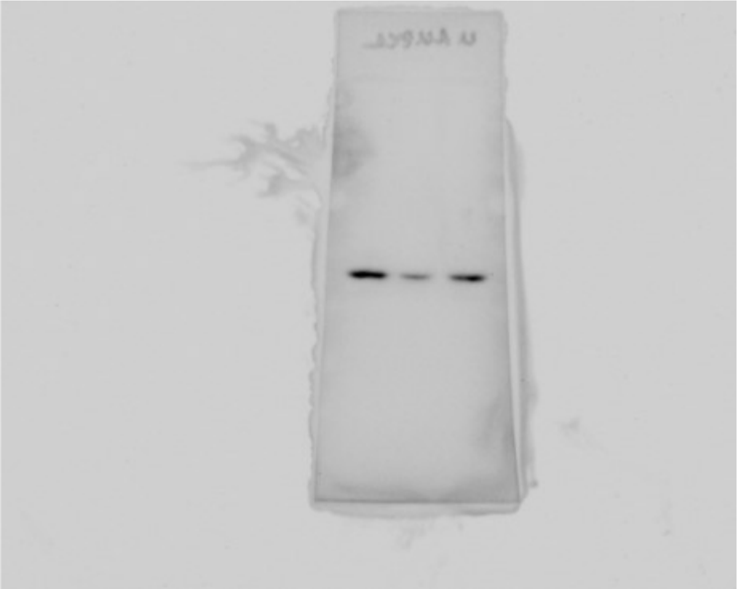

Fig. 4b

# U251-MG – images with protein ladder

Cyclin E1

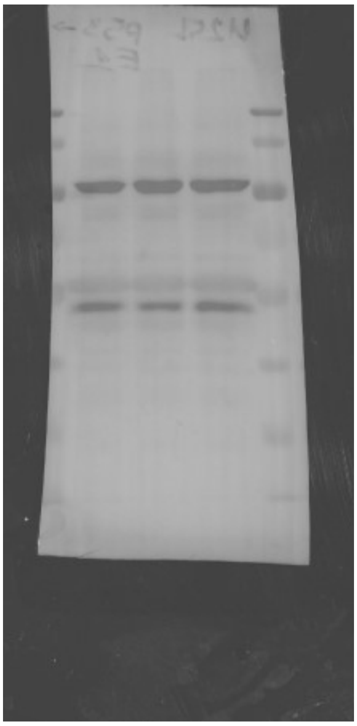

Cyclin E2

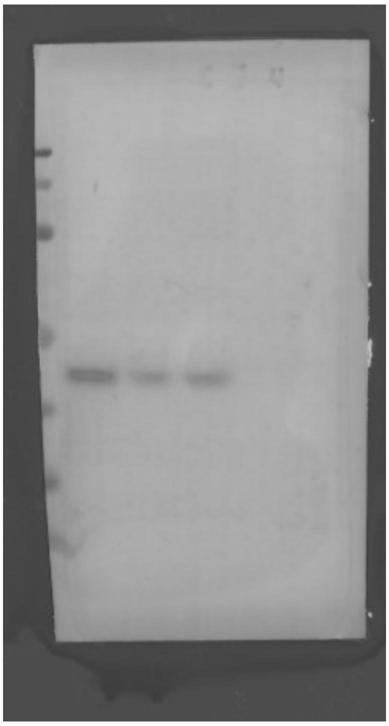

Cyclin D1

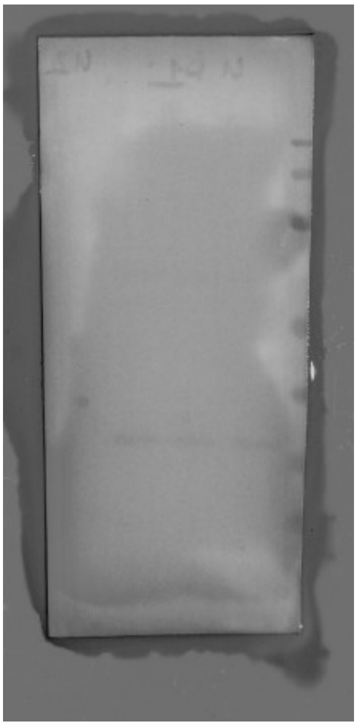

Cyclin A2

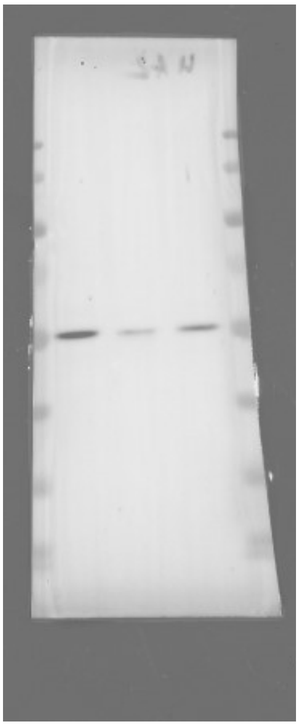

Cyclin B1

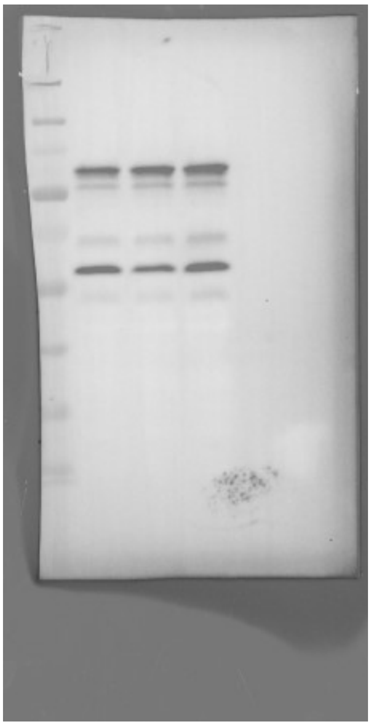

Aurora A kinase

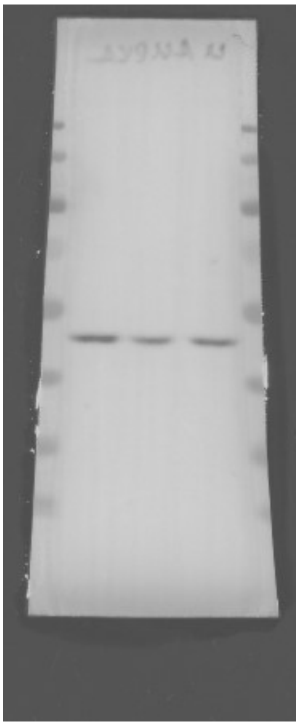

PageRuler Prestained Protein Ladder 10 to 180 kDa
was used (ThermoFisher Scientific, cat. 26616)

Fig. 4b

# U251-MG – cropped and adjusted

Cyclin E1

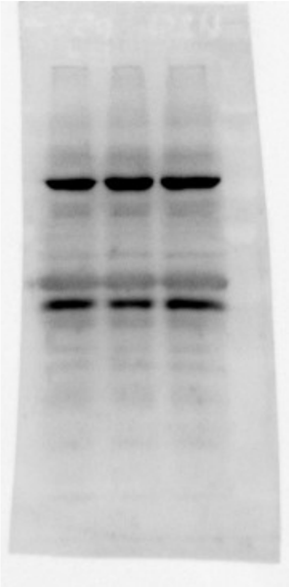

Cyclin E2

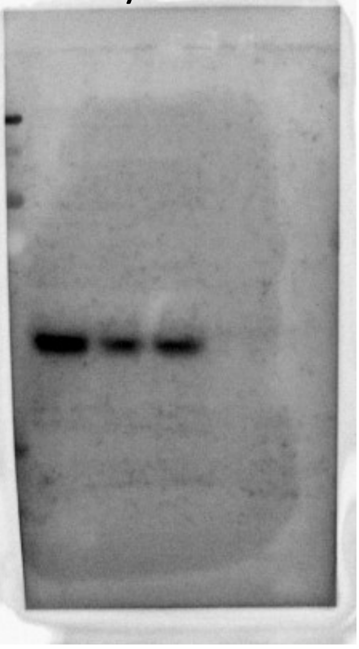

Cyclin D1

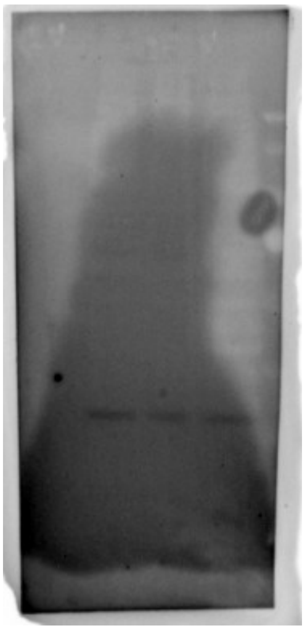

Cyclin A2

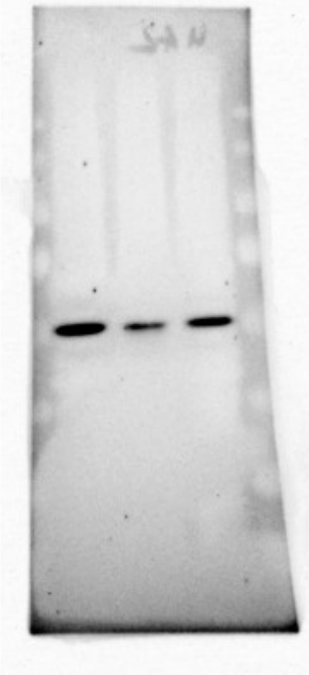

Cyclin B1

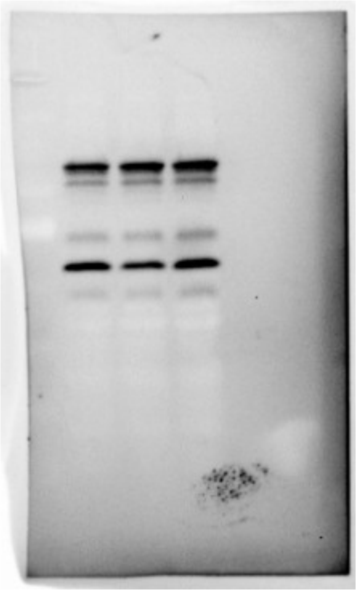

Aurora A kinase

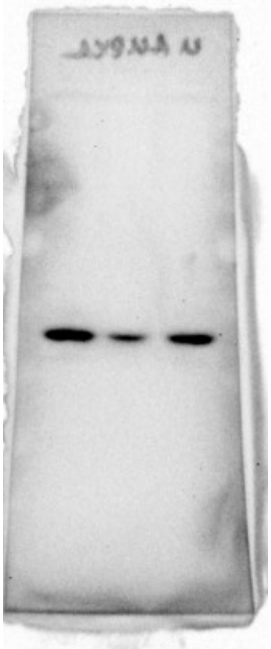

Fig. 4b

# U251-MG – Total protein

Cyclin E1

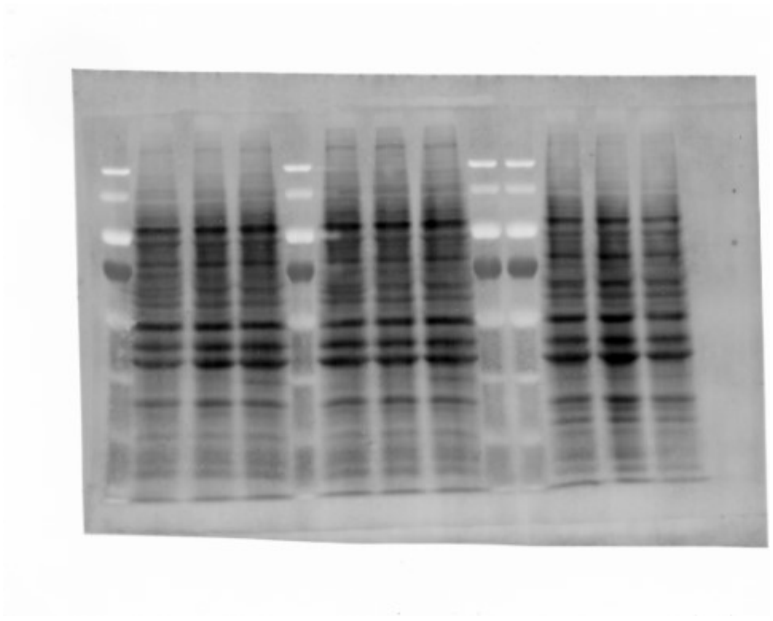

Cyclin E2

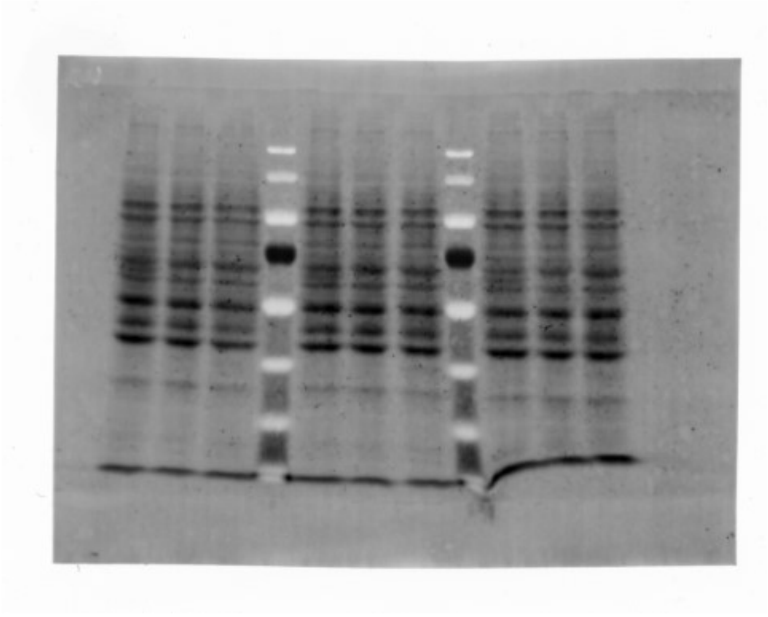

Cyclin D1

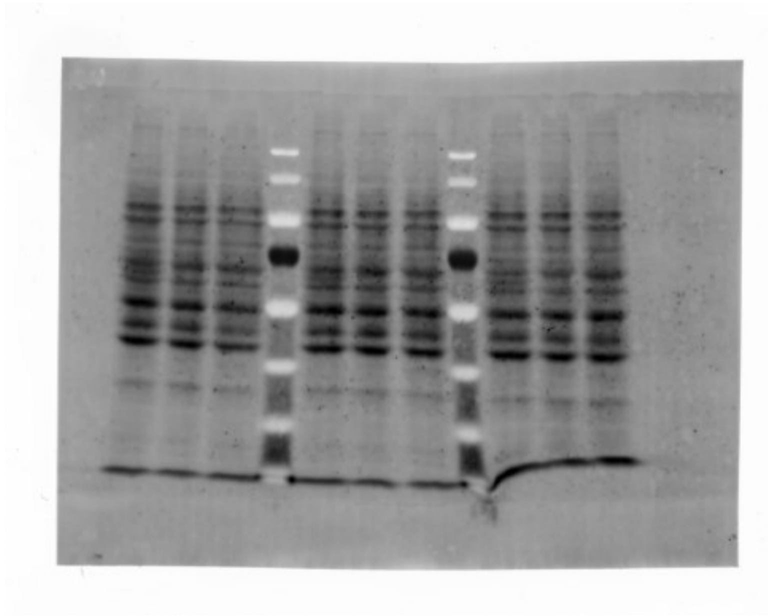

Cyclin A2

Aurora A kinase

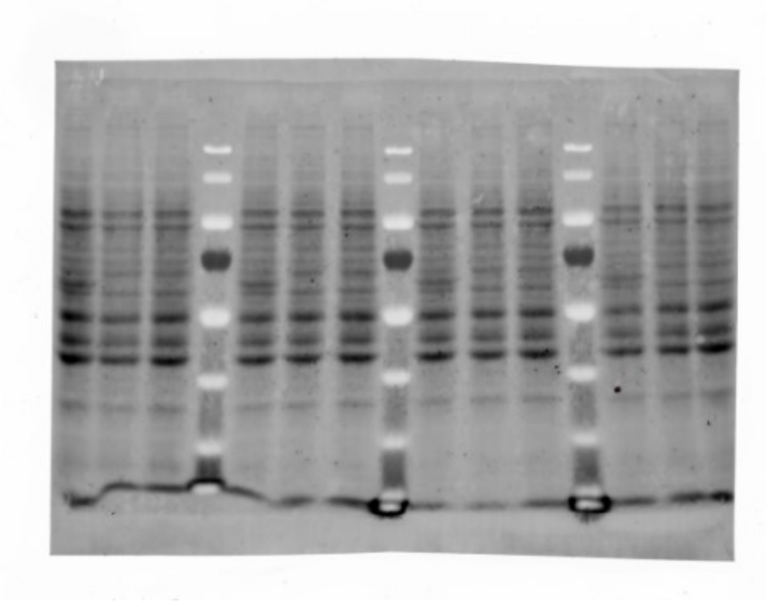

Cyclin B1

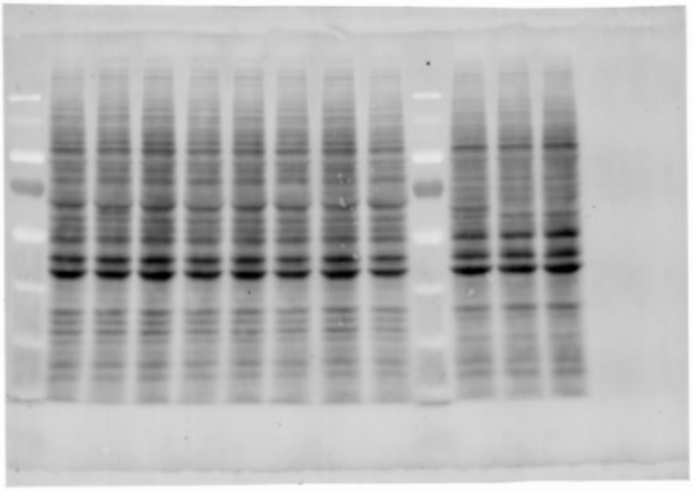

Fig. 4c

U87-MG

Cropped and adjusted

FLAG

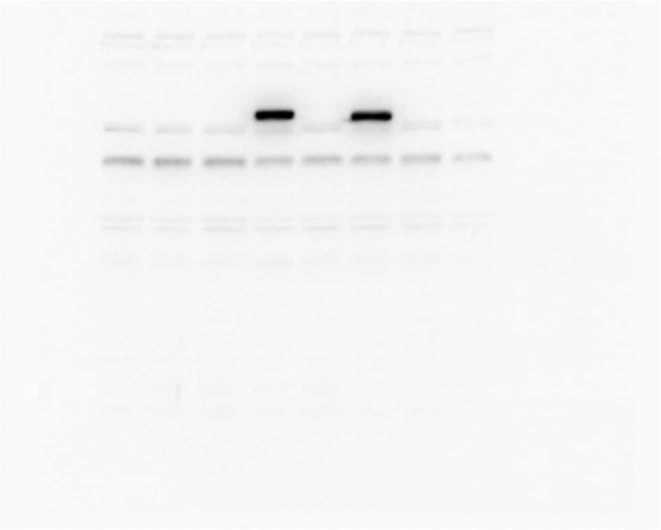

Oversaturated image with visible borders

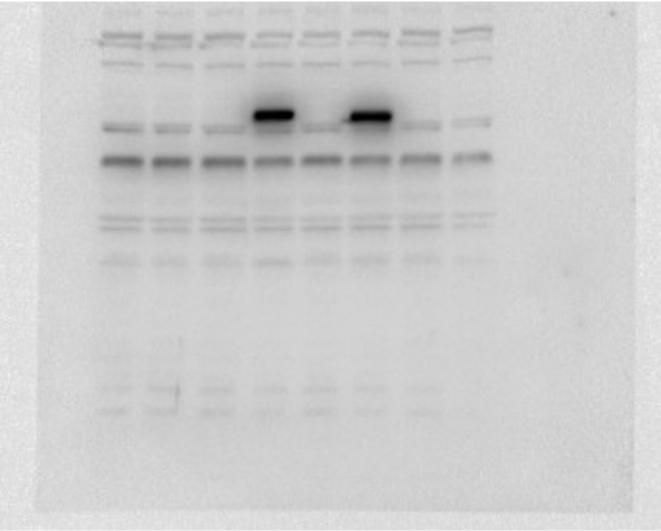

Original

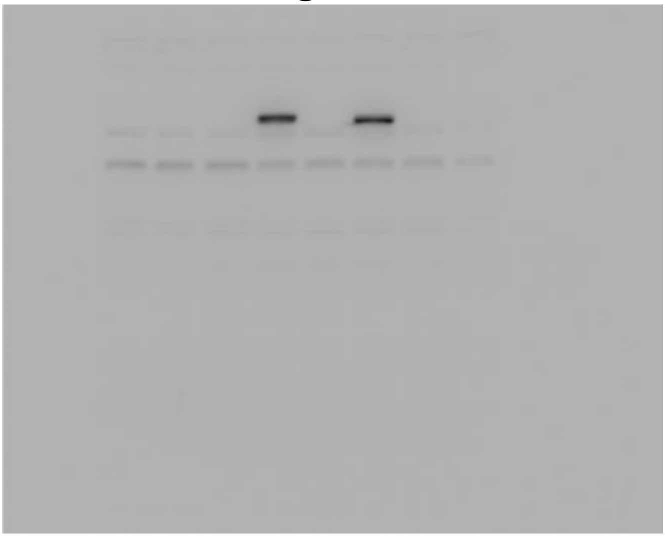

Image with protein ladder

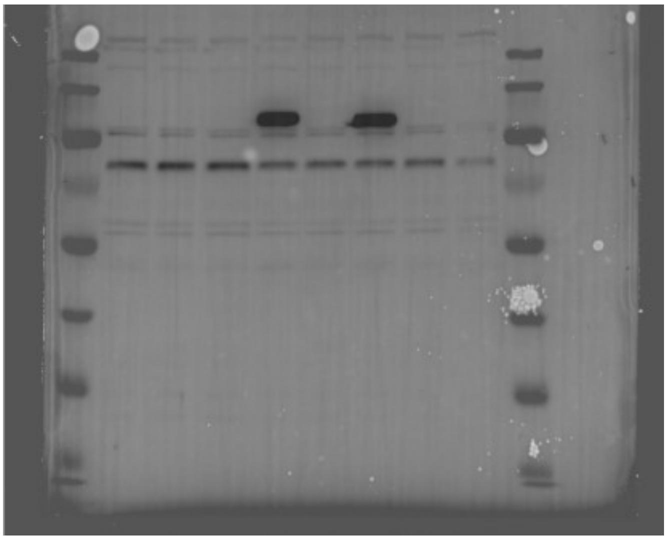

PageRuler Prestained Protein Ladder 10 to 180 kDa was used (ThermoFisher Scientific, cat. 26616)

Fig. 4c

U251-MG

Cropped and adjusted

FLAG

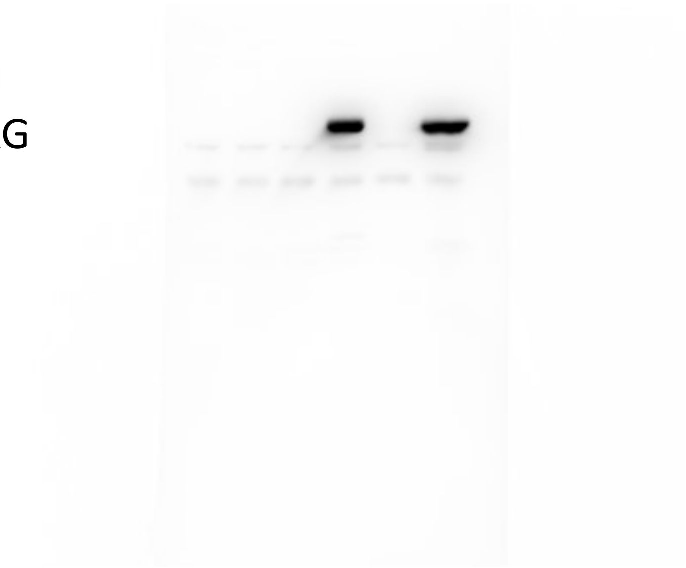

Oversaturated image with visible borders

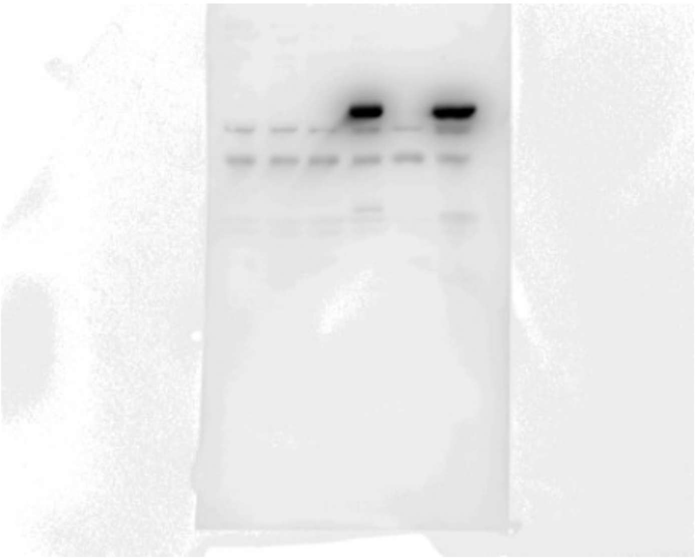

Original

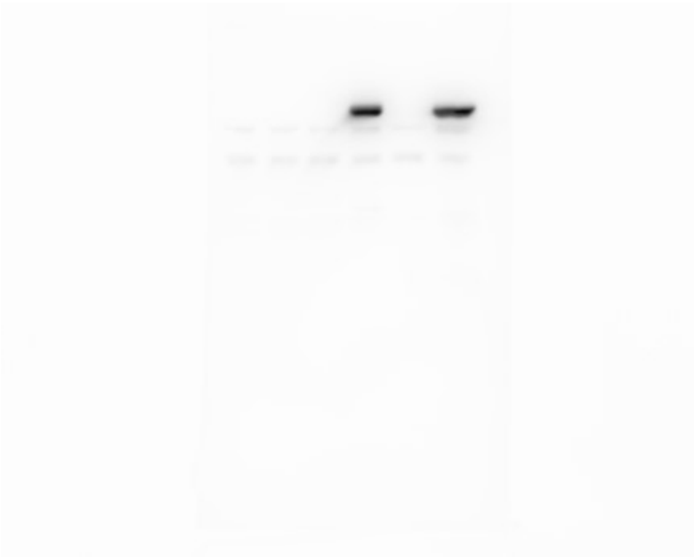

Image with protein ladder

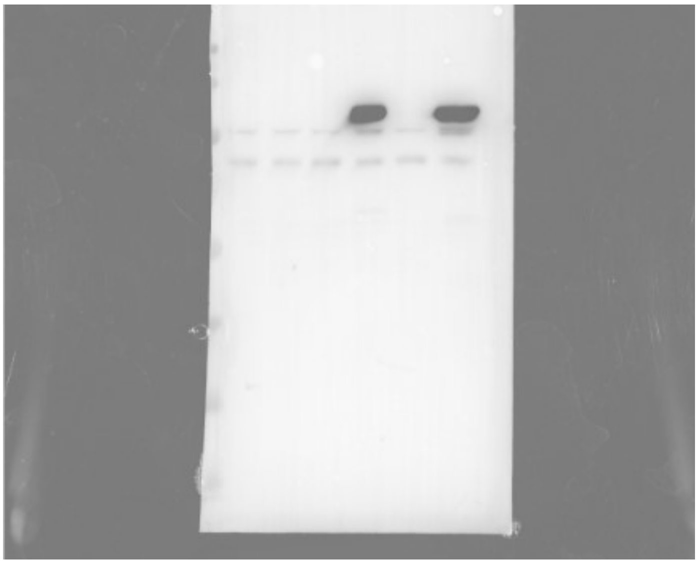

PageRuler Prestained Protein Ladder 10 to 180 kDa was used (ThermoFisher Scientific, cat. 26616)

Fig. 4c

U87-MG

Total protein

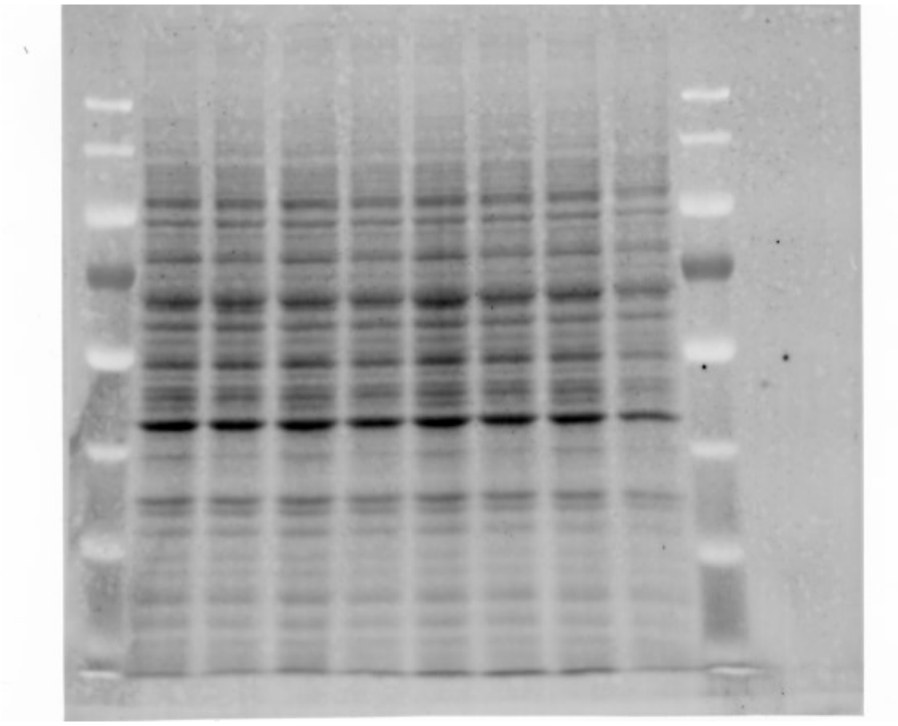

U251-MG

Total protein

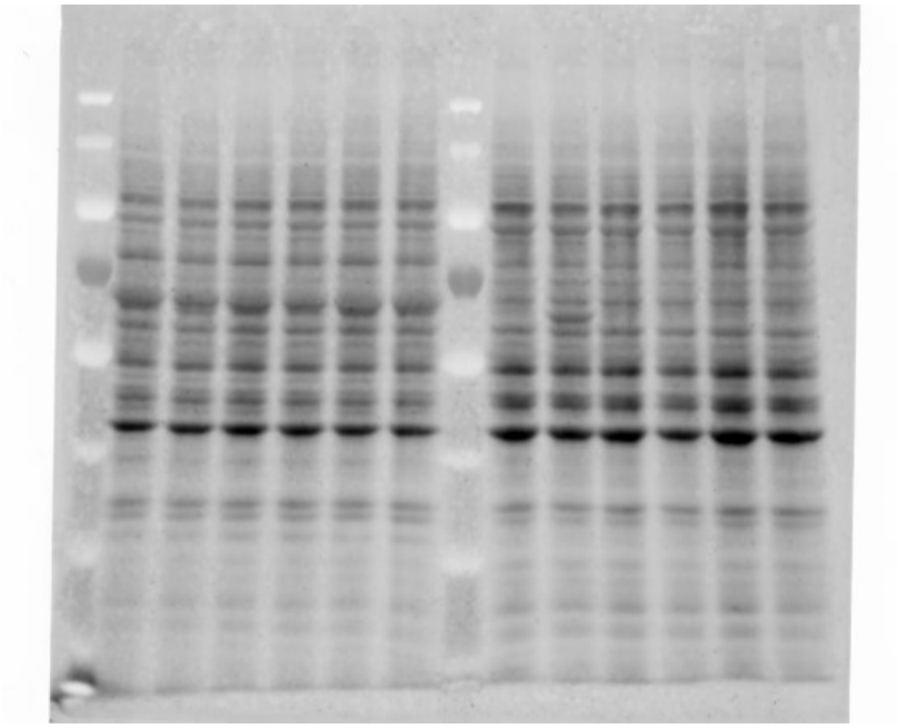

Supplement: Supplementary file 1 — Supplementary Information 1. [file 41598_2024_51809_MOESM1_ESM.pdf]
